# Continuous and Intermittent Artificial Gravity as a Countermeasure to the Cognitive Effects of 60 Days of Head-Down Tilt Bed Rest

**DOI:** 10.3389/fphys.2021.643854

**Published:** 2021-03-17

**Authors:** Mathias Basner, David F. Dinges, Kia Howard, Tyler M. Moore, Ruben C. Gur, Christian Mühl, Alexander C. Stahn

**Affiliations:** ^1^Division of Sleep and Chronobiology, Department of Psychiatry, Perelman School of Medicine at the University of Pennsylvania, Philadelphia, PA, United States; ^2^Brain Behavior Laboratory, Department of Psychiatry, Perelman School of Medicine at the University of Pennsylvania, Philadelphia, PA, United States; ^3^Department of Sleep and Human Factors Research, Institute of Aerospace Medicine, German Aerospace Center (DLR), Cologne, Germany

**Keywords:** cognition, spaceflight, performance, microgravity, bed rest, emotion recognition

## Abstract

Environmental and psychological stressors can adversely affect astronaut cognitive performance in space. This study used a 6° head-down tilt bed rest (HDBR) paradigm to simulate some of the physiologic changes induced by microgravity. Twenty-four participants (mean ± SD age 33.3 ± 9.2 years, *N* = 16 men) spent 60 consecutive days in strict HDBR. They were studied in three groups of eight subjects each. One group served as Control, whereas the other two groups received either a continuous or intermittent artificial gravity (AG) countermeasure of 30 min centrifugation daily (1 *g* acceleration at the center of mass and 2 *g* at the feet). Participants performed all 10 tests of NASA’s Cognition battery and a brief alertness and mood survey repeatedly before, during, and after the HDBR period. Test scores were adjusted for practice and stimulus set difficulty effects. A modest but statistically significant slowing across a range of cognitive domains was found in all three groups during HDBR compared to baseline, most consistently for sensorimotor speed, whereas accuracy was unaffected. These changes were observed early during HDBR and did not further worsen or improve with increasing time in HDBR, except for emotion recognition performance. With increasing time spent in HDBR, participants required longer time to decide which facial emotion was expressed. They were also more likely to select categories with negative valence over categories with neutral or positive valence. Except for workload, which was rated lower in the Control group, continuous or intermittent AG did not modify the effect of HDBR on cognitive performance or subjective responses. Participants expressed several negative survey responses during HDBR relative to baseline, and some of the responses further deteriorated during recovery, which highlights the importance of adequate medical and psychological support during extended duration HDBR studies. In conclusion, 60 days of HDBR were associated with moderate cognitive slowing and changes in emotion recognition performance, but these effects were not mitigated by either continuous or intermittent exposure to AG for 30 min daily.

## Introduction

Sustained high levels of astronaut cognitive performance are a prerequisite of successful human spaceflight. Several environmental, operational, physiologic, and psychological stressors related to living in the isolated, confined, and extreme spaceflight environment may adversely affect cognitive performance, and thereby pose risks to astronaut safety and health (Strangman et al., 2014; Basner et al., 2015; Stahn et al., 2019). Microgravity is one prominent stressor that has been implicated in the development of ocular and vision changes in spaceflight [spaceflight-associated neuro-ocular syndrome (SANS); Laurie et al., 2019; Marshall-Goebel et al., 2019a; Roberts et al., 2020]. Microgravity induces a physical body unloading and a headward fluid shift (Marshall-Goebel et al., 2019b). Structural brain changes observed in astronauts after return from International Space Station (ISS) missions have included an upward shift of the brain (Roberts et al., 2017; Lee et al., 2019; Roberts et al., 2019), changes in gray matter volume (Koppelmans et al., 2016), increased white matter in the cerebellum (Jillings et al., 2020), and cerebrospinal fluid volume increases in the third and lateral ventricles (Alperin et al., 2017; Roberts et al., 2017; Van Ombergen et al., 2019; Jillings et al., 2020; Kramer et al., 2020). The functional consequences of these physiologic and anatomical changes remain largely unknown (Roberts et al., 2019).

In addition to research performed mostly in low Earth orbit, international space agencies use ground-based analogs to investigate the effects of common spaceflight stressors on human physiology and performance. Head-down tilt bed rest (HDBR) has been used for at least 50 years as a ground-based analog for microgravity-induced physiologic changes (Pavy-Le Traon et al., 2007). Findings of studies investigating the effects of HDBR on cognitive performance have been inconclusive thus far, likely due to the diversity of cognitive test batteries used, protocol differences [e.g., exposure to stressors, degrees of head-down tilt (HDT), and duration], practice effect confounds, circadian time of testing, low sample size, and inadequate control groups (Lipnicki and Gunga, 2009). A few studies have investigated changes in cognitive performance induced by HDBR since the seminal review of Lipnicki and Gunga (2009). Rao et al. (2014) found that, while risk-taking behavior was not affected by 45 days of HDBR, functional MRI (fMRI) task activation patterns changed. A missing control group complicates the interpretation of the findings. In a 70-day HDBR study, Yuan et al. (2016) found a lower counting accuracy on a dual task, as well as a brain activation increase for dual tasking in the HDBR group, which implies that more neurocognitive control was needed for dual task execution during HDBR. Lipnicki et al. (2009) found no difference in Iowa Gambling Task performance induced by 51 days of HDBR. However, in contrast to ambulatory controls, HDBR subjects failed to adapt their card selection strategy as the task progressed. A study by Friedl-Werner et al. (2020) found that, compared to a group of subjects that received high-intensity interval training five to six times weekly during a 60-day HDBR study, the non-exercise control group demonstrated an increased BOLD signal in the left hippocampus and parahippocampal gyrus, while mnemonic performance on an episodic memory task did not differ between groups. This was interpreted as higher neuronal efficiency in the training group during memory encoding and retrieval.

Artificial gravity (AG) has been proposed as a countermeasure to the adverse physiologic effects induced by microgravity (Clement et al., 2015). It can be achieved either by rotating a spacecraft or station, or by centrifugation of the astronaut. Whereas neither have been implemented in spaceflight to date, the concept and its benefits are intriguing. Research on the effects of AG countermeasures during HDBR is scarce. One study investigated the effects of an AG countermeasure on spatial orientation of eight subjects undergoing 21 days of 6° HDBR (Moore et al., 2010). This study found a significant increase in error on a subjective visual vertical task for 48 h post bed rest. Another study investigated the effects of artificial gravity on cognitive performance during 21 days of 6° HDBR in 15 subjects using NASA’s WinSCAT tool (Seaton et al., 2007). These investigators found more off-nominal WinSCAT scores in the AG group (1 h centrifugation per day) relative to the control group, and accuracy tended to be more affected than speed.To further elucidate the effects of AG on general cognitive performance in HDBR, 24 subjects underwent 60 days of 6° HDBR in this study, eight of them exposed to continuous AG for 30 min daily, eight of them exposed to intermittent AG for 30 min daily, while the remaining eight served as controls without AG countermeasure. Participants performed NASA’s Cognition test battery and a brief alertness and mood survey repeatedly before, during, and after the HDBR period.

## Materials And Methods

### Study Design and Participants

This report includes data from a study on the effects of continuous and intermittent artificial gravity on participants spending 60 consecutive days in strict 6° HDBR performed at the :envihab at the German Aerospace Center (DLR) in Cologne, Germany, a research facility that allows for investigating up to 12 subjects concurrently under controlled environmental conditions. The study was titled Artificial Gravity Bed Rest – European Space Agency (AGBRESA). Study participants were randomly assigned to one of three groups consisting of *N* = 8 participants each, all of them undergoing 60 days of strict 6° HDBR: (1) Control group: no Artificial Gravity intervention; (2) continuous Artificial Gravity (cAG) group: one continuous 30-min bout of centrifugation daily; and (3) intermittent Artificial Gravity (iAG) group: six 5-min bouts of centrifugation with 3 min rest between bouts daily (see centrifugation protocol section below for details). Participants were pseudo-randomly assigned to groups in the first campaign. Due to three women dropping out in campaign 2, subsequent replacement was based on demographic balancing particularly with regards to sex. The 24 participants had an average age of 33.3 ± 9.2 years (range 23–54 years) and 14 (66.7%) were male. The three experimental groups did not differ significantly on age or sex (Supplementary Table S1).

Participants were recruited by the DLR. Study eligibility criteria included age between 24 and 55 years, non-smokers, body mass index between 19 and 30 kg/m^2^, no elevated risk of thrombosis, no recent history of bone fractures, and no history of chronic pain, hypertension, hyperlipidemia, arthritis, diabetes, obesity, hepatic disease, eye conditions, or a calcium/bone metabolism disorder. Subjects were screened to ensure that they were psychologically healthy before participation. They were empaneled 14 days before the start of the HDBR period (for baseline data acquisition) and discharged 14 days after the end of the HDBR period (for recovery phase data acquisition). The HDT position was continuously maintained throughout the course of the HDBR period. A specially designed neck support was allowed when subjects were on their sides during sleep, although many chose not to use it (see Laurie et al., 2020 for pictures). Subjects participated in several scientific investigations with a focus on SANS that were scheduled throughout the day, interrupted by meals that reflected a controlled diet. Participants were provided a daily 8-h sleep opportunity between 22:30 and 6:30. They were compensated for participating in the study, which was approved by the local ethics committee (Ärztekammer Nordrhein) and by the Institutional Review Board of NASA Johnson Space Center. Subjects provided written informed consent before participation and were allowed to discontinue the study at any time. The study was registered at the German Clinical Trials Register (DRKS) under #DRKS00015677.

### Centrifugation Protocol

Participants remained at 6° HDT at all times during transport to and from the 3.8 m radius short-arm centrifuge without using their leg muscles. Subjects were oriented radially in supine position on the centrifuge arm with their head toward the center of rotation and feet resting against a force plate. The centrifugation protocol included: acceleration at 5°/s^2^ for 32–33 s until target rotation speed was achieved followed by rotation at constant velocity (on average 30.5 rpm; with exposures of 1 *g* at participants’ estimated center of mass and 2 *g* at the feet) for either 30 min (cAG) or 5 min, with a 3-min rest, repeated six times (iAG). Deceleration was at 5°/s^2^. Participants were instructed not to move their head, relax their leg muscles, and to remain calm. All centrifugation runs were conducted between 09:00 h and 19:00 h, and the time of the day of the AG runs (morning vs. afternoon) were counterbalanced within subject. This was done to avoid any systematic effects of circadian variation on countermeasure tolerance and/or efficacy, but the timing of the centrifuge runs also necessarily changed daily to allow for scientific testing that needed to be scheduled before the commencing of centrifugation on any given bed rest day. Participant safety was guaranteed through continuous medical monitoring.

### Cognition Test Battery and Cognition Outcome Variables

The following description of the Cognition battery was modified from Basner et al. (2015, 2017). Cognition contains a subset of tests from a widely used and validated neurocognitive battery, the Penn Computerized Neurocognitive Battery (PennCNB; Gur et al., 2010, 2012; Moore et al., 2014), and a number of additional tests. Cognition emphasizes tests that have either been used extensively in spaceflight or that assess cognitive domains of particular interest in spaceflight (such as spatial orientation, emotion recognition, and risk decision making; Lim and Dinges, 2008; Usui et al., 2009). The 10 Cognition tests were modified to reflect the high aptitude and motivation of astronauts. They assess a range of cognitive domains, and the brain regions primarily recruited by each test have been previously established. Importantly, Cognition has 15 unique stimulus sets (i.e., versions) that allow for repeated administration without the need to re-use stimulus sets. Six tests have unique stimulus sets, while the remaining four tests (Motor Praxis, Line Orientation, Digit Symbol Substitution, and Psychomotor Vigilance) randomly generate stimuli each time the test is administered. A detailed overview of Cognition can be found in Basner et al. (2015).

Analyses concentrated on one main accuracy and one main speed outcome for each Cognition test. Congruent with descriptions in Basner et al. (2020a), all accuracy outcomes ranged from 0 to 100% with 100% representing best possible performance. For all speed outcomes, lower values reflect shorter response times and thus higher speed. Average response time (milliseconds) was the speed outcome for all tests except the PVT (see below). Percentage correct was the accuracy outcome for five *Cognition* tests. The accuracy outcomes for the other tests are described for each test below. All outcomes were corrected for practice and stimulus set difficulty effects according to Basner et al. (2020a) based on an administration interval of 5 days or less before statistical analyses. All Cognition outcomes were also z-transformed based on the average and SD of baseline performance scores (administrations 9, 7, and 6 days before bed rest) across study subjects and conditions (i.e., the average and SD used for z-transformation were based on 3*24 = 72 scores). Summary scores for accuracy and speed were calculated by averaging across z-transformed scores within the accuracy and speed domain, respectively. Speed summary scores were multiplied by −1 so that higher scores reflected higher speed. An efficiency score was calculated by averaging the accuracy and speed summary (z) scores. In the following paragraphs, we provide a brief description of each of the 10 Cognition tests. The tests were always performed in the order listed below, starting with stimulus set 1 and sequentially progressing through the 15 stimulus sets.

The **Motor Praxis Task (MP)** was administered as the first test to ensure that participants had sufficient command of the computer interface. It is a measure of sensorimotor speed (Gur et al., 2001). Participants were instructed to click on squares that appear randomly on the screen, with each successive square smaller and thus more difficult to track. Performance was assessed by the speed with which participants click each square. For the MP accuracy outcome, the distance from the center of each square (in pixels) was averaged across all responses. The center of the square translated to 100% accuracy, 50 pixels or more away from the center translated to an accuracy score of 0%, with linear scaling between 0 and 50 pixels.

The **Visual Object Learning Test (VOLT)** assessed participant memory for complex figures (Glahn et al., 1997). Participants were asked to memorize 10 sequentially displayed 3D Euclidean shapes. Later, they were presented with 20 such objects, half of them from the learning set and half new. Participants were instructed to decide for each object whether they had seen it before or not, and how confident they were in their decision (“definitely” or “probably”).

The **Fractal 2-Back (F2B)** is a nonverbal variant of the Letter 2-Back. *N*-back tasks have become standard probes of the working memory system and activate canonical working memory brain areas (Ragland et al., 2002). The F2B consists of the sequential presentation of a set of figures (fractals), each potentially repeated multiple times. Participants are instructed to respond when the current stimulus matches the stimulus displayed two figures ago. The current implementation used 62 consecutive stimuli. For the F2B accuracy outcome, the percentage correct for matches and non-matches was averaged to avoid subjects achieving good accuracy scores even if they never hit the spacebar.

The **Abstract Matching (AM)** test is a validated measure of the abstraction and flexibility components of executive function, including an ability to discern general rules from specific instances (Glahn et al., 2000). The test paradigm presents subjects with two pairs of objects at the bottom left and right of the screen, varied on perceptual dimensions (e.g., color and shape). Subjects are presented with a target object in the upper middle of the screen that they have to classify as belonging more with one of the two pairs, based on a set of implicit, abstract rules. The current implementation used 30 consecutive stimuli.

The **Line Orientation Test (LOT)** is a measure of spatial orientation and derived from the well-validated Judgment of Line Orientation Test (Benton et al., 1978). The LOT format consists of presenting two lines at a time, one stationary while the other could be rotated by clicking an arrow. Participants can rotate the movable line until they perceive it to be parallel to the stationary line. The implementation used in this study had 12 consecutive line pairs that varied in length and orientation. The LOT accuracy measure was calculated as 3 minus the average number of clicks off, which was then divided by 3 (lines are rotated with 2^0^ per click on the LOT; subjects are on average ~0.8 clicks off). For tests with more than 3 clicks off on average, the accuracy score was set to 0%.

The **Emotion Recognition Task (ERT)** is a measure of facial emotion recognition (Gur et al., 2010). It presents subjects with photographs of professional actors (adults of varying age and ethnicity) portraying emotional facial expressions of varying types and intensities (biased toward lower intensities, and with the prevalence of emotion categories balanced within each version of the test). Subjects are given a set of emotion labels (happy, sad, angry, fearful, and no emotion) and have to select the label that correctly describes the expressed emotion. The implementation used in the study had 40 consecutive stimuli, with 8 stimuli each representing one of the five emotion categories. Stimuli that loaded negatively in an Item Response Theory (IRT) analysis were excluded for the calculation of both ERT speed and ERT accuracy (see Basner et al., 2020a for a list of excluded stimuli).

The **Matrix Reasoning Test (MRT)** is a measure of abstract reasoning and consists of increasingly difficult pattern matching tasks (Gur et al., 2001). It is analogous to Raven Progressive Matrices (Raven, 1965), recruits prefrontal, parietal, and temporal cortices (Perfetti et al., 2009) and is based on a well-known measure of general intelligence. The test consists of a series of patterns, overlaid on a grid. One element from the grid is missing and the participant has to select the element that fits the pattern from a set of alternative options. The implementation used in the study applied 12 consecutive stimuli. Stimuli that loaded negatively in an IRT analysis were excluded for the calculation of both MRT speed and MRT accuracy (see Basner et al., 2020a for a list of excluded stimuli).

The **Digit-Symbol Substitution Task (DSST)** is a computerized adaptation of a paradigm used in the Wechsler Adult Intelligence Scale (WAIS-III; Usui et al., 2009). The DSST required the participant to refer to a displayed legend relating each of the digits one through nine to specific symbols. One of the nine symbols appears on the screen and the participant has to select the corresponding number as quickly as possible. The test duration is fixed at 90 s, and the legend key is randomly re-assigned with each administration.

The **Balloon Analog Risk Test (BART)** is a validated assessment of risk-taking behavior (Lejuez et al., 2002). The BART requires participants to either inflate an animated balloon or stop inflating and collect a reward. Participants are rewarded in proportion to the final size of each balloon, but a balloon pops after a hidden number of pumps that changes across stimuli, in which case the reward is voided. The implementation used in the study had 30 consecutive stimuli. The average tendency of balloons to pop is varied systematically between test administrations. This variation requires subjects to adjust the level of risk based on the behavior of the balloons. It prevents subjects from identifying a strategy during the first administrations of the battery and carrying it through to later administrations. For each pump on the BART, a value of 1 divided by the number of possible pumps across all 30 balloons was added to the BART Risk Score. This Risk Score, therefore, takes into account that different sets of balloons popped at different inflation rates. We list BART risk-taking as an accuracy outcome despite the fact that it inherently measures risk-taking. For this reason, it was not included in calculating the accuracy summary score across cognitive domains (see above).

The **Psychomotor Vigilance Test (PVT)** records reaction times (RT) to visual stimuli that occur at random inter-stimulus intervals (Basner and Dinges, 2011). The PVT is a sensitive measure of vigilant attention, and sensitive to acute and chronic sleep deprivation as well as circadian misalignment (Barger et al., 2014). Subjects are instructed to monitor a box on the screen, and press the space bar once a millisecond counter appears in the box and starts incrementing. The reaction time is displayed for 1 s. Subjects are instructed to be as fast as possible without hitting the spacebar in the absence of a stimulus (i.e., false starts or errors of commission). In the current study, Cognition contained a validated 3-min brief PVT-B with 2–5 s inter-stimulus intervals and a 355 ms lapse threshold (Basner et al., 2011). For the PVT, 10 minus reciprocal response time (1/RT) was used as the speed outcome, as this metric was shown to be a superior outcome for the PVT relative to average RT (Basner and Dinges, 2011). The PVT accuracy score was calculated as 1 − [(# of Lapses + # of False Starts)/(Number of Stimuli + # of False Starts)]. Any response time not falling between the false start threshold (100 ms) and the lapse threshold (355 ms) thus decreased accuracy on the PVT.

The Cognition software administered a brief survey before each administration of the test battery. Participants entered the time they tried to fall asleep and woke up, which was used as an estimate of their sleep duration. They then indicated their current status on the following 13 11-point Likert scales (anchors are provided in parenthesis after each question; the middle point was labeled neutral): (1) What was the quality of your sleep? (good-poor); (2) What was today’s workload? (very high-very low); How are you feeling right now? (3; not sleepy at all-very sleepy); (4; happy-unhappy); (5; sick-healthy); (6; energetic-physically exhausted); (7; mentally sharp-mentally fatigued); (8; not stressed at all-very stressed); (9; tired-fresh, ready to go); (10; very depressed-not depressed at all); (11; very bored-not bored at all); (12; not lonely at all-very lonely); and (13) What is your current everyday life like? (very monotonous-not monotonous at all). For analysis purposes, items 2, 5, 9, 10, 11, and 13 were inverted so that high scores always reflected more negative responses.

### Cognition Procedures

Participants first watched a standardized familiarization video. They then performed the full Cognition battery twice for task familiarization 13 and 11 days before the start for the HDBR period. They were required to perform a brief practice version immediately before each test during the first familiarization bout (except for the VOLT and BART, which do not have practice versions). Cognition was performed three more times on days 9, 7, and 6 before bed rest. These administrations served as baseline. Cognition then was performed on days 1, 3, 5, 14, 28, 42, and 57 after the initiation of the bed rest period. Finally, Cognition was administered on days 1, 5, and 12 during the recovery period following bed rest.

Cognition (version 3.0.9, using the version 2 ERT with 40 stimuli) was administered on Dell laptop computers (12.5″ screen diagonal, 1,366 × 768 resolution) calibrated for timing accuracy in the afternoon (mean ± SD of administration time across all study periods: 17:24 ± 0:33). It was performed in the seated upright position before and after the bed rest period. For testing in the HDT position, laptops were mounted vertically on an adjustable swivel arm and positioned in chest-height in front of the participants (see Supplementary Figure S1). Participants used the laptop’s track pad and integrated mouse button to operate the arrow on the screen.

### Statistical Analyses

Data were analyzed with SAS (SAS Institute, Carey, NC, United States; version 9.4). Linear mixed effect models with random intercepts were used to account for the fact that test data were clustered within participants. Survey data were treated as continuous for analysis purposes (Leung, 2011). All models were adjusted for sex and age (continuous variable). Furthermore, all models were adjusted for baseline performance, with the exception of models for subjective outcomes and sleep duration (unless otherwise noted). Values of *p* were adjusted for multiple testing according to the false discovery rate method (Benjamini and Hochberg, 1995) for the 23 Cognition outcomes (one standard speed and accuracy outcome for each test plus accuracy, speed, and efficiency across tests; i.e., *N* = 23 comparisons) and for the 13 subjective outcomes and sleep duration (i.e., *N* = 13 comparisons). We provide both unadjusted values of *p* and confidence intervals as well as the alpha level that survived adjustment (i.e., *p* < 0.05, *p* < 0.01, *p* < 0.001, and *p* < 0.0001).

The following analyses were performed on both cognitive performance and self-report outcomes: (1) The difference in baseline assessments between the three experimental groups was assessed; (2) marginal means were estimated for the Control and AG groups during the HDT phase and the recovery phase using observed marginal means for sex, age, and baseline performance. As z-transformation was performed using baseline data only, estimated marginal means reflect the difference of cognitive test scores during HDT/recovery to baseline cognitive test scores, adjusted for potential differences in baseline performance between the three groups; (3) the cAG group and the iAG group were contrasted to the Control group separately for the bed rest and the recovery phase; (4) it was investigated whether assessments changed linearly with time in HDBR, and whether the slope differed significantly between groups (i.e., group*time interaction). Model 4 was the only model that allowed for random intercepts and random slopes (unstructured covariance).

## Results

Data were extracted and visualized for each subject. Seven out of 3,600 expected test bouts (data 99.8% complete) were excluded from data analysis, six due to subject non-compliance, and one due to technical difficulties. The three experimental groups did not differ significantly at baseline in terms of cognitive performance, sleep duration, or survey responses (Supplementary Table S1). Self-reported sleep duration averaged 7.54 h in the Control group, 7.61 h in the cAG group, and 7.55 h in the iAG group, respectively. Subjects in all three groups reported moderate levels of tiredness, sleepiness, sleep quality, mental fatigue, physical exhaustion, and workload; low levels of monotony, boredom, loneliness, depression, and stress; and high levels of health and happiness at baseline.

### Head-Down Tilt Bed Rest Effects

**Figure 1:**
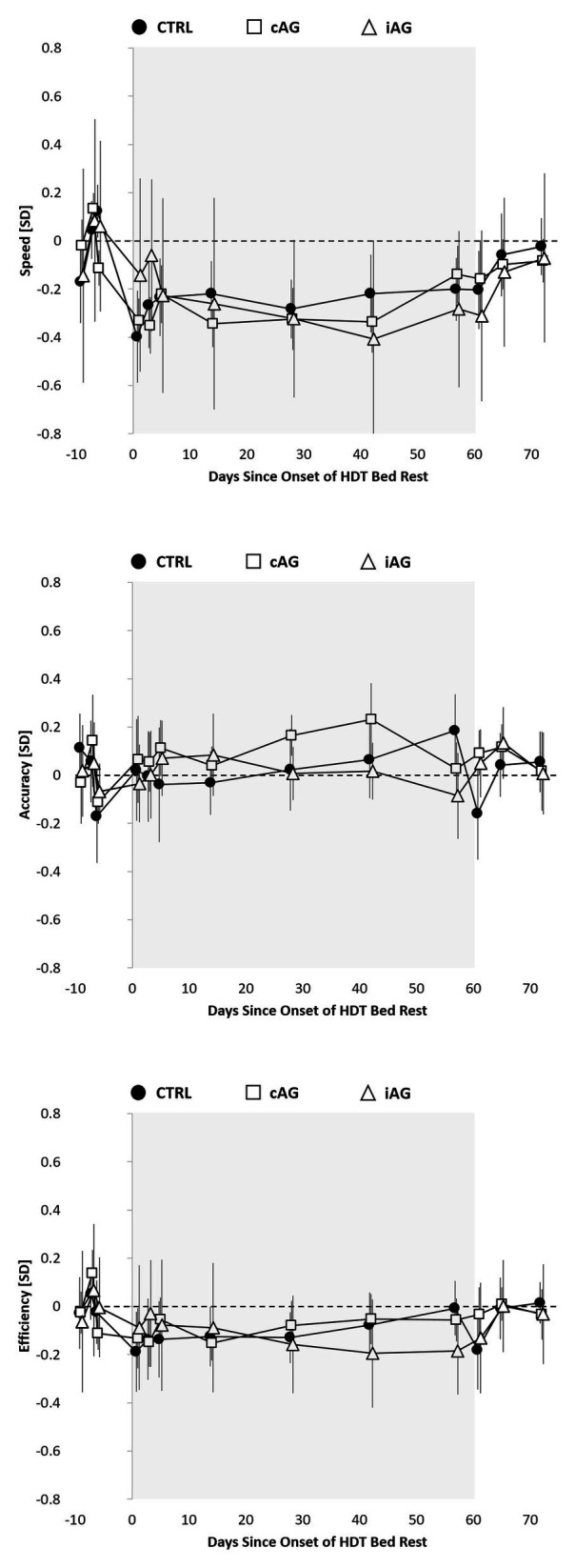
Cognitive speed, accuracy, and efficiency across cognitive domains relative to the 60-day head-down tilt (HDT) bed rest period (gray background) for the Control group (black circles), continuous artificial gravity group (cAG; white squares), and intermittent artificial gravity group (iAG; white triangles). Estimates reflect unadjusted means z-transformed based on baseline (pre-HDT) performance within each of the 10 Cognition tests and then averaged across tests. To reflect the analytical approach (adjusting for baseline performance), means were shifted within groups to reflect a pre-HDT baseline performance of 0 (zero).

**Figure 2:**
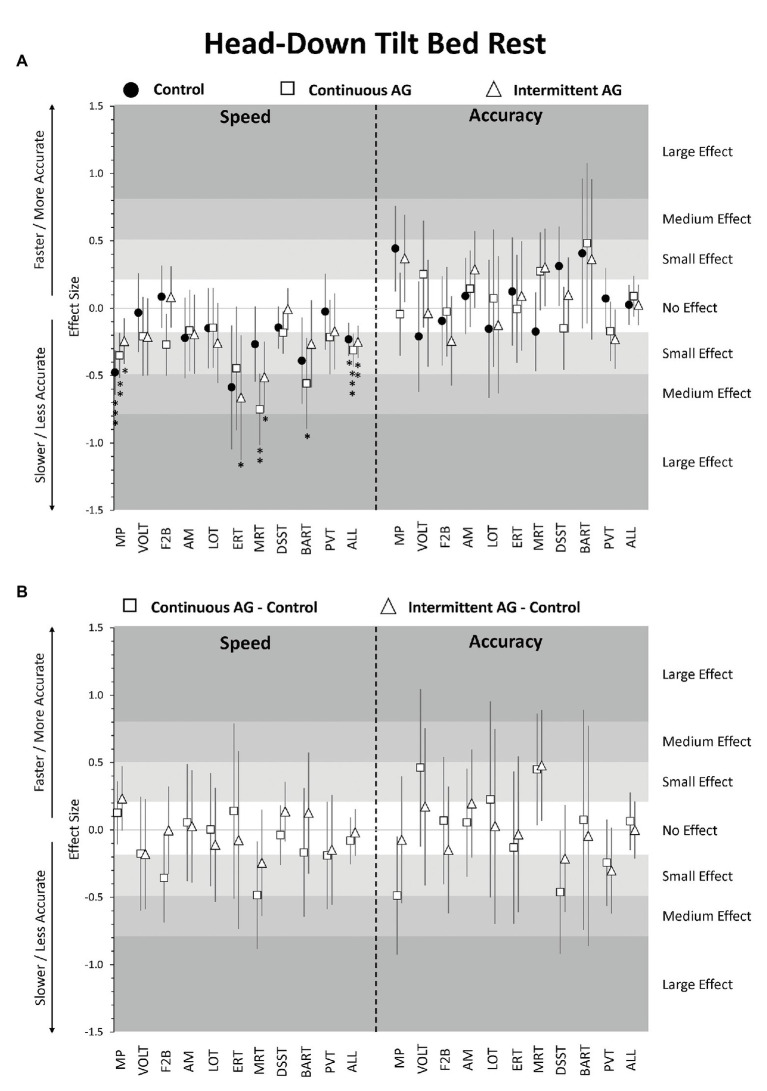
Change in cognitive performance in the head-down tilt (HDT) bed rest period relative to pre-HDT baseline. Estimates reflect z-scores based on the mean and SD of pre-HDT baseline performance. Error bars reflect unadjusted 95% confidence intervals. **(A)** Estimates for the Control group (black circles), cAG group (white squares), and iAG group (white triangles); **(B)** Estimates for the difference cAG-Control (squares) and iAG-Control (triangles); ^*^adjusted *p* < 0.05; ^**^adjusted *p* < 0.01; ^***^adjusted *p* < 0.001; MP, Motor Praxis; VOLT, Visual Object Learning Test; F2B, Fractal 2-Back; AM, Abstract Matching; LOT, Line Orientation Test; ERT, Emotion Recognition Test; MRT, Matrix Reasoning Test; DSST, Digit Symbol Substitution Test; BART, Balloon Analog Risk Test; PVT, Psychomotor Vigilance Test; ALL, scores averaged across cognitive domains.

Compared to baseline performance, there was a small but statistically significant decrease in speed across cognitive domains observed in all experimental groups (Control −0.23 SD, adjusted *p* < 0.05; cAG −0.31 SD, adjusted *p* < 0.001; iAG −0.25 SD, adjusted *p* < 0.01; Figures 1, 2; Supplementary Table S2). Accuracy across cognitive domains did not differ from baseline in any of the three groups (effect sizes <0.1 SD; all *p* > 0.22). Cognitive efficiency also did not differ relative to baseline in any of the groups after adjustment for multiple testing. Neither cognitive speed, accuracy nor efficiency differed significantly between the three groups (effect sizes ≤0.08; all *p* > 0.34; Supplementary Table S2).

Focusing on individual tests, speed was significantly slower on the MP in all three groups, BART speed was significantly lower in the cAG group only, and ERT and MRT speed was significantly lower in the iAG group only (Figure 2A; Supplementary Table S2). Accuracy did not differ significantly from baseline for any of the 10 tests in any of the three groups. Also, none of the speed or accuracy outcomes differed between the three groups for any of the 10 tests after adjusting for multiple testing (Figure 2B; Supplementary Table S2).

**Figure 3:**
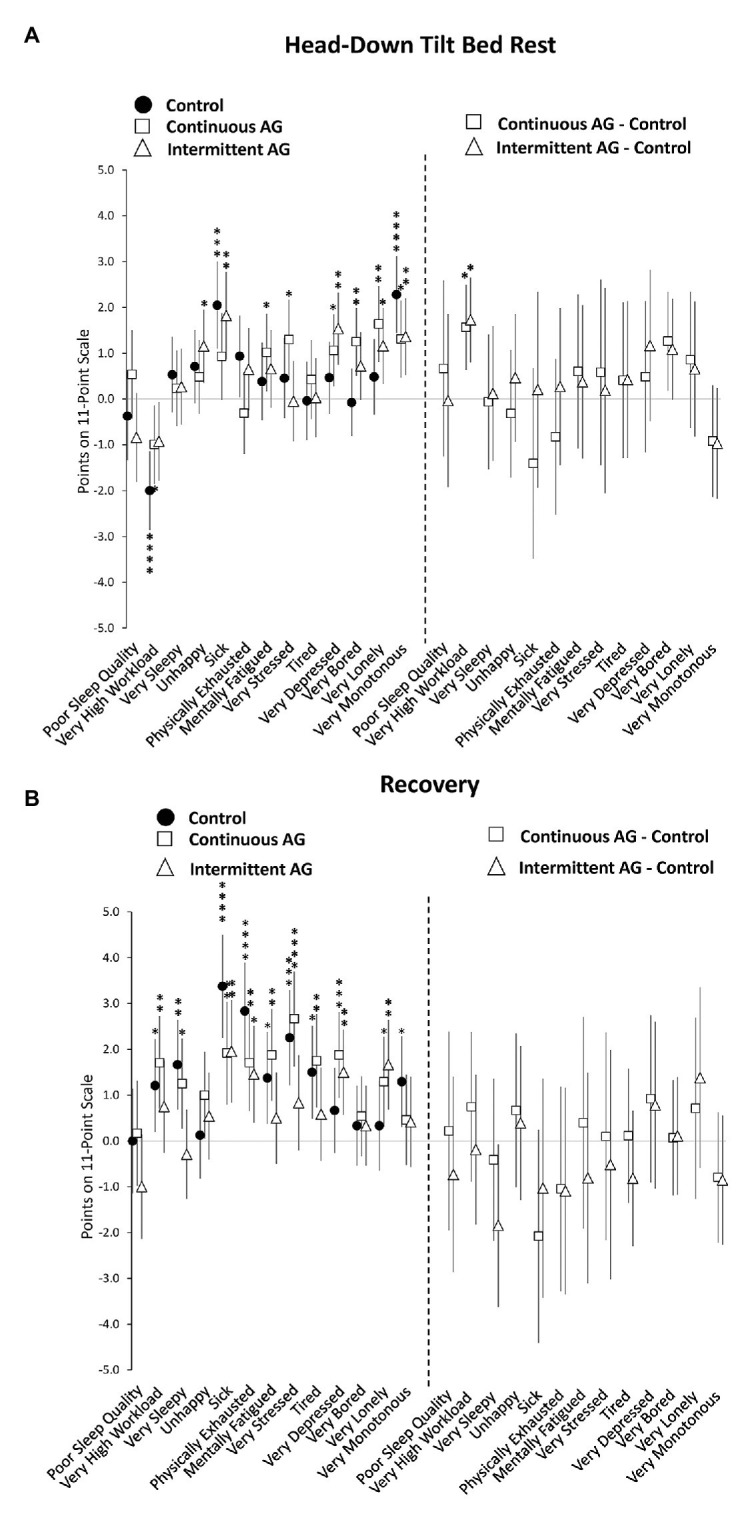
Change in survey responses during head-down tilt (HDT) bed rest **(A)** and post-HDT recovery **(B)** relative to pre-HDT baseline for the Control group (black circles), cAG group (white squares), and iAG group (white triangles). Estimates reflect points on an 11-point scale. For each variable, the negative response anchor is shown (e.g., “unhappy” and “very sleepy”). Positive scores reflect more negative assessments relative to baseline (graphs on the left) or control (graphs on the right). Error bars reflect unadjusted 95% confidence intervals. ^*^adjusted *p* < 0.05; ^**^adjusted *p* < 0.01; ^***^adjusted *p* < 0.001; ^****^adjusted *p* < 0.0001.

Self-reported sleep duration did not differ significantly between bed rest and baseline periods for any of the three experimental groups (Supplementary Table S2). Analyses of the survey responses showed significantly lower levels of happiness in the iAG group, significantly higher levels of sickness in the Control group and iAG group, significantly higher levels of mental fatigue and stress in the cAG group, significantly higher levels of depression, boredom, and loneliness in the cAG group and iAG group, and significantly higher levels of monotony in all three groups during the HDBR period compared to baseline (Figure 3A; SupplementaryTable S2). However, the only reliable difference between the three groups was a significantly higher rating of workload in the cAG and iAG groups relative to Control (Figure 3A; Supplementary Table S2).

**Figure 4:**
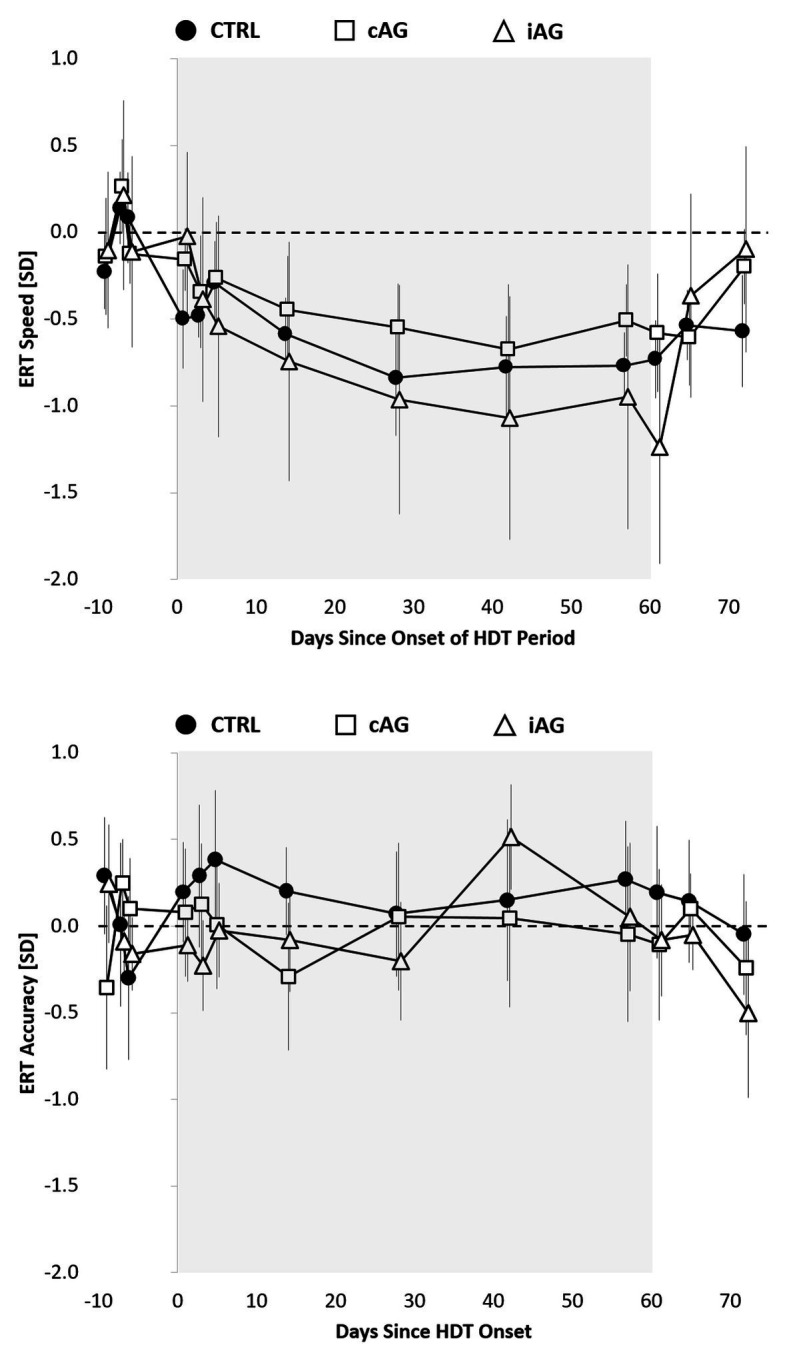
Speed and accuracy on the Emotion Recognition Test (ERT) relative to the 60-day HDT bed rest period (gray background) for the control group (black circles), cAG group (white squares), and iAG group (white triangles). Estimates reflect unadjusted means (SEs) z-transformed based on baseline (pre-HDT) performance. To reflect the analytical approach (adjusting for baseline performance), means were shifted within groups to reflect a pre-HDT baseline performance of 0 (zero).

Except for ERT speed, none of the other cognitive test outcomes, survey responses, or sleep duration changed significantly with days in HDBR (all adjusted *p* > 0.05; Supplementary Table S4; Supplementary Figures S2–S10). Also, time in HDBR slopes did not differ significantly between groups (all adjusted *p* > 0.05 for time*group interaction; Supplementary Table S4). Speed on the ERT decreased significantly with time in HDBR (*β* = −0.009 SD per day in HDBR; adjusted *p* < 0.01; Supplementary Table S4; Figure 4). The decline in response speed on the ERT was consistently observed across the three experimental groups (unadjusted *p* = 0.2580 for time*group interaction). ERT accuracy did not change significantly with days in HDBR. An in-depth analysis of ERT responses showed that, with increasing time spent in HDBR, subjects were significantly more likely to rate faces angry (adjusted *p* < 0.01) and significantly less likely to rate them happy (adjusted *p* < 0.05) or neutral (adjusted *p* < 0.05; Supplementary Table S4).

### Recovery Effects

**Figure 5:**
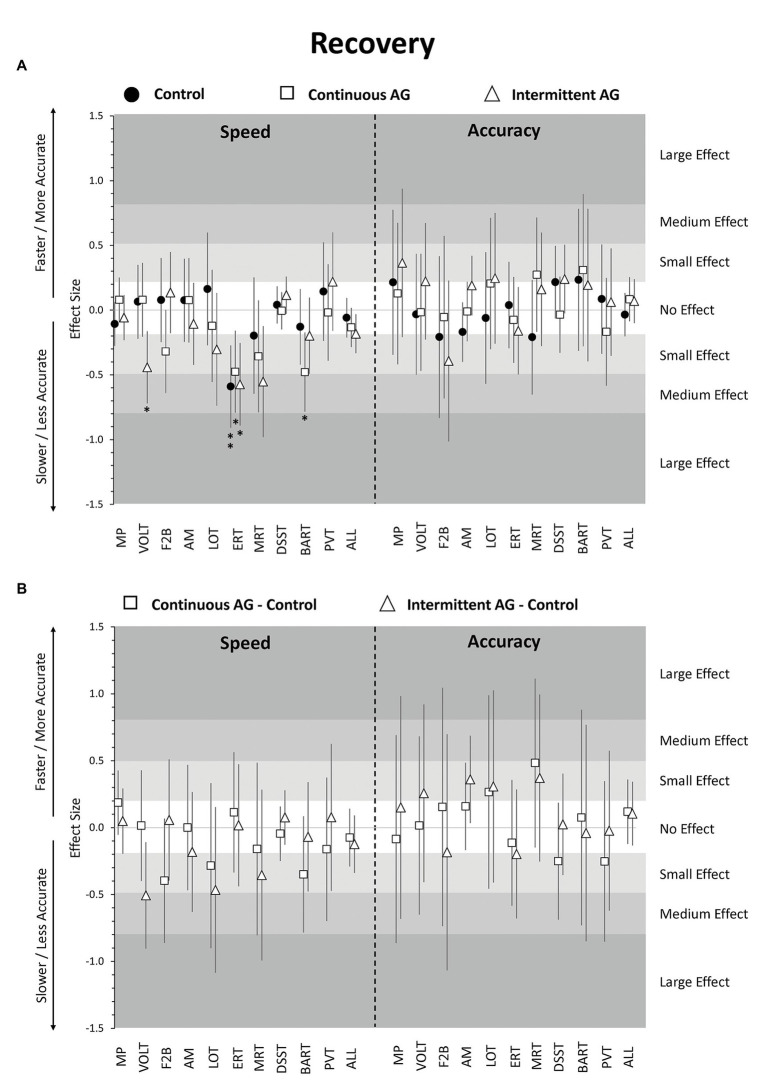
Change in cognitive performance in post-HDT recovery period relative to pre-HDT baseline. Estimates reflect z-scores based on the mean and SD of pre-HDT baseline performance. Error bars reflect unadjusted 95% confidence intervals. **(A)** Estimates for the Control group (black circles), cAG group (white squares), and iAG group (white triangles); **(B)** Estimates for the difference cAG-Control (white squares) and iAG-Control (white triangles); ^*^adjusted *p* < 0.05; ^**^adjusted *p* < 0.01; MP, Motor Praxis; VOLT, Visual Object Learning Test; F2B, Fractal 2-Back; AM, Abstract Matching; LOT, Line Orientation Test; ERT, Emotion Recognition Test; MRT, Matrix Reasoning Test; DSST, Digit Symbol Substitution Test; BART, Balloon Analog Risk Test; PVT, Psychomotor Vigilance Test; ALL, scores averaged across cognitive domains.

Point estimates for cognitive speed across cognitive domains were negative for the three experimental groups during the recovery period relative to baseline, but effect sizes were <0.2 SD and non-significant after adjustment for multiple testing. Raw data plots (Figure 1) suggest a gradual return of cognitive speed to baseline, with similar or even slower performance on the first ambulatory day during recovery relative to the last test administration on HDBR day 57. Accuracy and efficiency across cognitive domains did not differ during recovery from baseline for any of the three groups. Neither cognitive speed, accuracy, nor efficiency across cognitive domains differed significantly between any of the three groups during the recovery phase (Figures 1, 5; Supplementary Table S3).

Focusing on individual tests, ERT speed was significantly lower in all three groups relative to baseline with effect sizes ranging from −0.59 to −0.48 (all adjusted *p* < 0.05; Figure 5A; Supplementary Table S3). In addition, VOLT speed was significantly lower in the iAG group, while BART speed was significantly lower in the cAG group. None of the other tests differed from baseline in either of the three groups for both speed and accuracy. Also, none of the speed and accuracy outcomes differed between the three groups for any of the 10 Cognition tests in the recovery phase after adjustment for multiple testing (Figure 5B; Supplementary Table S3).

Sleep duration did not differ significantly between recovery and baseline periods for the Control group or the cAG group, but was 0.57 h shorter during recovery in the iAG group (adjusted *p* < 0.01; Supplementary Table S3). Several subjective ratings differed significantly from baseline during recovery. All three groups rated themselves significantly less healthy and significantly more physically exhausted (Figure 3A; Supplementary Table S3). Workload, sleepiness, mental fatigue, stress, and tiredness were rated significantly higher in the Control group and cAG group only. Depression and loneliness were rated significantly higher in the cAG group and iAG group only. Finally, only the Control group rated higher levels of monotony compared to baseline. However, neither sleep duration nor survey responses differed significantly between the three groups during the recovery period (all adjusted *p* > 0.05; Figure 3B; Supplementary Table S3).

## Discussion

This study investigated the effects of a 60-day 6° HDBR period with and without an artificial gravity countermeasure on cognitive performance across a range of cognitive performance domains. A small but reliable slowing of cognitive speed across a range of cognitive domains was found in all three experimental groups with the onset of HDBR. Twenty-eight out of 30 (i.e., 93.3%) individual test speed point estimates across the three groups were negative. The slowing was most consistently observed for MP, the only Cognition test with significantly slower response speed during HDT relative to baseline in each of the three experimental groups. MP is a measure of sensorimotor speed that probes the sensorimotor cortex. Both spaceflight (Roberts et al., 2017; Lee et al., 2019; Roberts et al., 2019) and bed rest studies (Roberts et al., 2015) have demonstrated an upward shift of the brain with increased brain tissue density at the vertex, which includes the somatosensory cortex and could be the cause for the observed slowing. Some of the reductions seen in the other nine tests also may be explained by reduced sensorimotor speed, because all Cognition tests have a sensorimotor component.

Other HDBR studies have likewise observed a response slowing for selected cognitive domains (Lipnicki and Gunga, 2009; Liu et al., 2012), which could be related to an increase in delta and theta EEG frequencies induced by HDBR and interpreted as signs of cortical inhibition (Vaitl et al., 1996). This finding is also consistent with a 30-day HDBR study (titled VaPER) performed at DLR :envihab, where CO_2_ levels were increased to ~3.73 mmHg during the bed rest period (Basner et al., 2021). The fact that response slowing is not a more consistent finding across HDBR studies may be attributed to practice effect confounds or missing ambulatory controls. In addition, to our knowledge, strict HDT has rarely been enforced in past HDBR studies, which could have played a role.

A recent study investigating associations between Cognition performance and complex 6° of freedom docking performance found that speed on AM, LOT, and especially DSST were associated with high docking performance (Basner et al., 2020b). While point estimates indicate an HDBR-induced response slowing on all three tests, effect sizes were small and did not differ significantly from baseline, suggesting that the observed changes may have had limited impact on operationally relevant performance. However, additional HDBR studies with operationally relevant tasks need to verify that operational performance is not affected relevantly.

Accuracy was unaffected during HDBR, both across domains and for the 10 individual Cognition tests. This finding suggests that participants were able to maintain stable accuracy levels by slowing down. Accordingly, cognitive efficiency across tests also did not differ during HDBR from baseline.

In comparisons between the three experimental groups, there was no evidence for an effect of either continuous or intermittent artificial gravity on cognitive speed or accuracy. Indeed, the consistency in performance among the three groups was remarkable. While the study may have been underpowered to detect small differences between groups, none of the point estimates indicated even a medium effect size (>0.5 SD) for any difference between groups. This finding, therefore, suggests that a daily 30-min centrifugation protocol with exposures of 1 g at participants’ estimated center of mass and 2 g at the feet was not sufficient to mitigate the HDBR-induced effects on cognitive speed. Furthermore, it suggests that the exposure modality (continuous vs. intermittent AG) does not play a relevant role, at least, at this centrifugation intensity and duration. Ultimately, 30 min of centrifugation translate to only 2.1% of the 24-h day, and this exposure duration may simply be too short to mitigate the cognitive effects caused by prolonged HDBR. Future studies will be needed to determine whether different modes or longer durations of centrifugation are more effective in reducing the effects of HDBR on cognitive slowing.

We are aware of only a single other study that investigated the effects of artificial gravity on general cognitive performance during 21 days of 6° HDBR in 15 subjects using NASA’s WinSCAT tool (Seaton et al., 2007). These investigators found more off-nominal WinSCAT scores in the AG group (1 h centrifugation per day) relative to the control group, and accuracy tended to be more affected than speed. Comparable to our study, the length of time spent in bed rest was not associated with a change in cognitive function (WinSCAT does not probe emotion recognition).

With the exception of speed on the ERT, cognitive speed and accuracy did not change significantly with time in HDBR on any of the 10 Cognition tests – thus, any change observed initially during HDBR remained stable until HDBR day 57. This stability not only suggests that the changes induced by HDBR were instantaneous, but also that they neither ameliorated nor further deteriorated over a period of 60 days. Speed on the ERT decreased significantly with time in HDBR and the slope of change did not differ between the three experimental groups. In contrast, ERT accuracy did not change significantly with time in HDT. An in-depth analysis showed that subjects were also significantly less likely to rate faces as happy or neutral and more likely to rate them as angry with increasing time spent in HDT. These findings suggest that participants not only needed significantly more time with increasing time spent in HDBR to identify the correct emotion, but they also developed a response bias from responses of neutral or positive valence to responses of negative valence. The spaceflight relevance of a deterioration of emotional processing with increasing time in mission cannot be overstated, especially for exploration space missions, where astronauts will be confined to a small space with a small group of peers for a period of up to 3 years.

Previous research also found evidence for changes in emotional processing during a 30-day 6° HDBR study using event-related potentials (ERP), with an inhibition of P300 and late positive potential (LPP) components for emotional stimuli, but not neutral pictures, suggestive of emotional blunting (Brauns et al., 2019). Messerotti Benvenuti et al. (2013) likewise found emotional blunting in P300 and LPP components after only 3 h of 6° HDBR. In our study, participants stayed in separate rooms and had only sporadic contact with the study team. It is therefore, unclear whether the changes in emotional processing observed in this study were caused by prolonged periods of HDBR, low levels of human interaction, or both. Interestingly, neither ERT speed nor ERT accuracy declined during a 30-day HDBR study with elevated levels of ambient CO_2_ (~3.73 mmHg) performed by the same study team in the same research facility (Basner et al., 2021). It is, however, unclear whether the elevated CO_2_, the shorter HDBR duration, or other factors that differed from the study discussed here can explain this finding. Future HDBR studies should consider varying the degree of social isolation to disentangle the mechanisms involved in altered emotional processing.

The cognitive slowing observed in the bed rest period did not immediately return to baseline levels during recovery. Cognitive speed across domains was similar or even slightly lower on recovery day 1 compared to HDBR day 57, and then gradually recovered on recovery days 5 and 12. No evidence was found for a significant difference among the three experimental groups during the recovery period.

All study participants showed healthy survey responses before the HDBR period. During the HDBR period, several negative survey responses were observed, with participants feeling less healthy and expressing higher levels of depression, boredom, loneliness, and monotony. Subjective assessments indicated lower levels of workload in all three groups, but significantly more so in the Control group. Many of these negative survey responses further deteriorated during the recovery phase, especially ratings of sickness, physical exhaustion, mental fatigue, and stress, without a significant difference among experimental groups. These findings suggest a considerable psychological toll of spending 60 days in HDBR, including difficulties re-adapting to ambulatory conditions.

## Strengths and Limitations

To our knowledge, this is the first study investigating the effects on cognitive performance of a continuous and intermittent artificial gravity countermeasure during and after a 60-day HDBR period. The breadth of the Cognition test battery, the near completeness of the data, and the ability to adjust for practice and stimulus set effects are strengths of this study (Basner et al., 2020a). Practice effects in the absence of proper ambulatory controls may have restricted the interpretability of cognitive test data obtained in most bed rest studies performed to date (Lipnicki and Gunga, 2009). That HDT was strictly enforced is another strength of this study. Strict HDBR accurately replicates the sustained head-ward fluid shift that occurs in weightlessness and creates a consistent and uniform stimulus. This consistency seems especially relevant for studies that investigate neurostructural and functional effects of HDBR.

However, the study also had several limitations. First and foremost, HDBR is a spaceflight analog, and as such an imperfect replication of the conditions caused by microgravity and other stressors in spaceflight. Whereas the change in gravity vector is the most plausible explanation for the observed effects, we cannot rule out other contributing factors (e.g., performing the cognitive tests in an unusual body position). However, it was not possible to quantify the contribution of individual factors as they were perfectly confounded with the HDBR intervention. Similar limitations apply to cognitive testing in spaceflight. Also, as evidenced by the large 95% confidence intervals in Figure 2B, we were likely underpowered to find small or even medium effect sizes statistically significant in this between-subject design with a group size of *N* = 8. Larger studies are needed to more conclusively eliminate artificial gravity as an effective countermeasure for cognitive performance deficits induced by HDBR.

### Conclusion

This study found a small but statistically reliable slowing of cognitive performance across a range of cognitive functions induced by 60 days of 6° HDBR, most consistently for sensorimotor speed, whereas accuracy was unaffected. These changes were observed early during HDBR and neither deteriorated further nor improved with increasing time in HDBR. The only exception was the Emotion Recognition Test. After an initial drop in speed on HDBR day 1, subjects needed increasingly more time with longer time spent in HDBR to decide which facial emotion was displayed, and they also favored categories with negative valence over categories with neutral or positive valence. The success of long-duration space missions will critically depend on astronaut emotional health, and correctly reading each other’s facial expressions is an important part of this domain. Except for workload, which was assessed lower in the Control group relative to both artificial gravity groups, this study found no evidence for an effect of either continuous or intermittent artificial gravity on either cognitive performance or subjective responses. Participants expressed several negative survey responses during HDBR and some of them further deteriorated during the recovery phase, stressing the importance of adequate medical and psychological support during extended duration bed rest studies.

## Data Availability Statement

The datasets generated for this study can be found by request at the NASA Life Science Data Archive (https://lsda.jsc.nasa.gov/).

## Ethics Statement

The studies involving human participants were reviewed and approved by Ärztekammer Nordrhein and the Institutional Review Board of Johnson Space Center. Participants provided their written informed consent to participate in this study.

## Author Contributions

MB and AS: study design. CM, MB, KH, and AS: data collection. KH and MB: database management. MB: data analysis, manuscript draft, and figures. MB, AS, DD, TM, RG, and CM: results interpretation. MB, AS, KH, DD, TM, RG, and CM: manuscript revisions. All authors contributed to the article and approved the submitted version.

### Conflict of Interest

The authors declare that the research was conducted in the absence of any commercial or financial relationships that could be construed as a potential conflict of interest.
