# Supplementary material for: Continuous and Intermittent Artificial Gravity as a Countermeasure to the Cognitive Effects of 60 Days of Head-Down Tilt Bed Rest
Source: Front Physiol. 2021 Mar 17;12:643854. doi: 10.3389/fphys.2021.643854 (PMC8009974; doi:10.3389/fphys.2021.643854)
Supplement: Supplementary file 1 [file Data_Sheet_1.pdf]

Supplementary Materials

Continuous and Intermittent Artificial Gravity as a Countermeasure to the Cognitive Effects of 60 Days of Head-Down Tilt Bed Rest

Mathias Basner,[1,*] David F. Dinges,[1] Kia Howard,[1] Tyler M. Moore,[2] Ruben C. Gur,[2] Christian Mühl,[3] Alexander C. Stahn[1]

[1] Division of Sleep and Chronobiology, Department of Psychiatry, Perelman School of Medicine at the University of Pennsylvania, Philadelphia, PA

[2] Brain Behavior Laboratory, Department of Psychiatry, Perelman School of Medicine at the University of Pennsylvania, Philadelphia, PA

[3] Department of Sleep and Human Factors Research, Institute of Aerospace Medicine, German Aerospace Center (DLR), Cologne, Germany

*** Correspondence:**
Mathias Basner, MD, PhD, MScEpi
Professor of Psychiatry
Perelman School of Medicine at the University of Pennsylvania
1019 Blockley Hall, 423 Guardian Drive
Philadelphia, PA 19104-6021, USA
Tel: +1 215 573-5866
Fax: +1 215 573-6410
basner@pennmedicine.upenn.edu

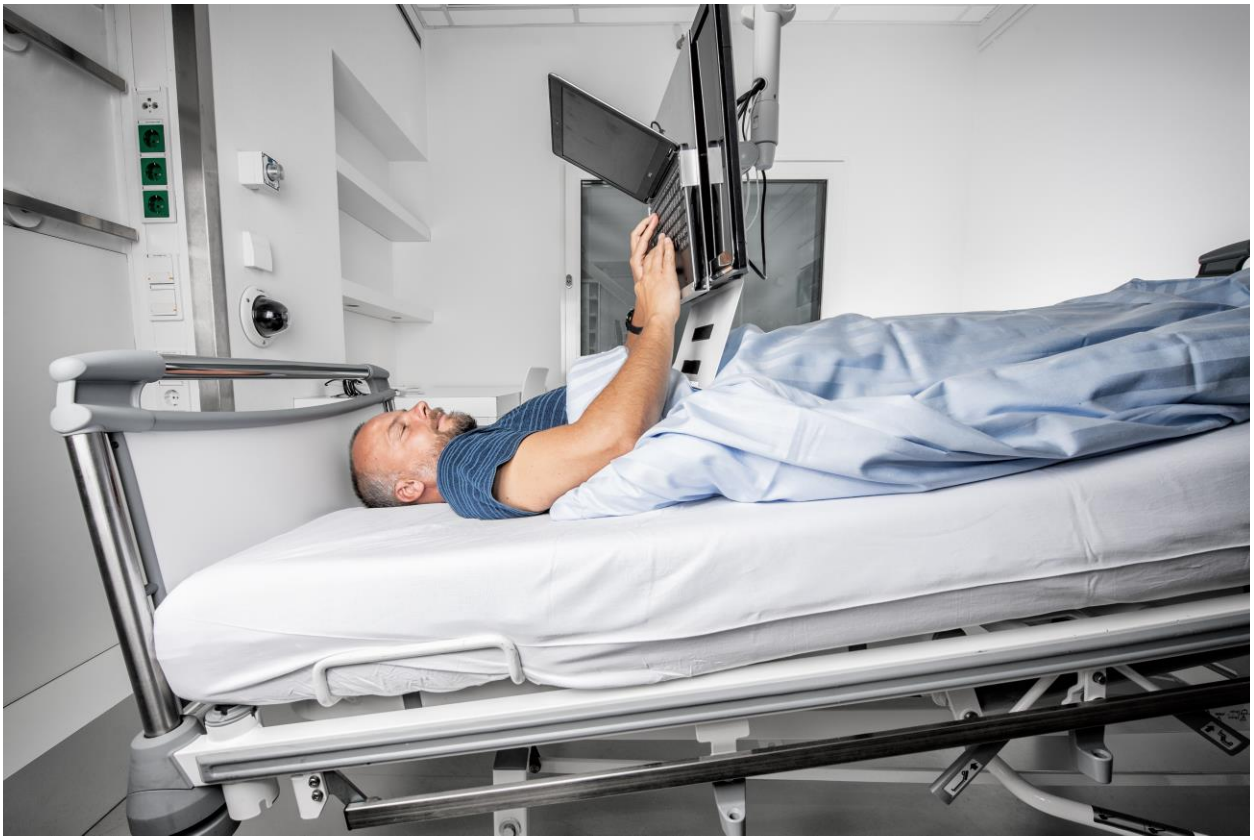

Figure S1: For testing in the HDT position, laptops were mounted vertically on an adjustable swivel arm and positioned in chest-height in front of the participants (source: DLR, with permission).

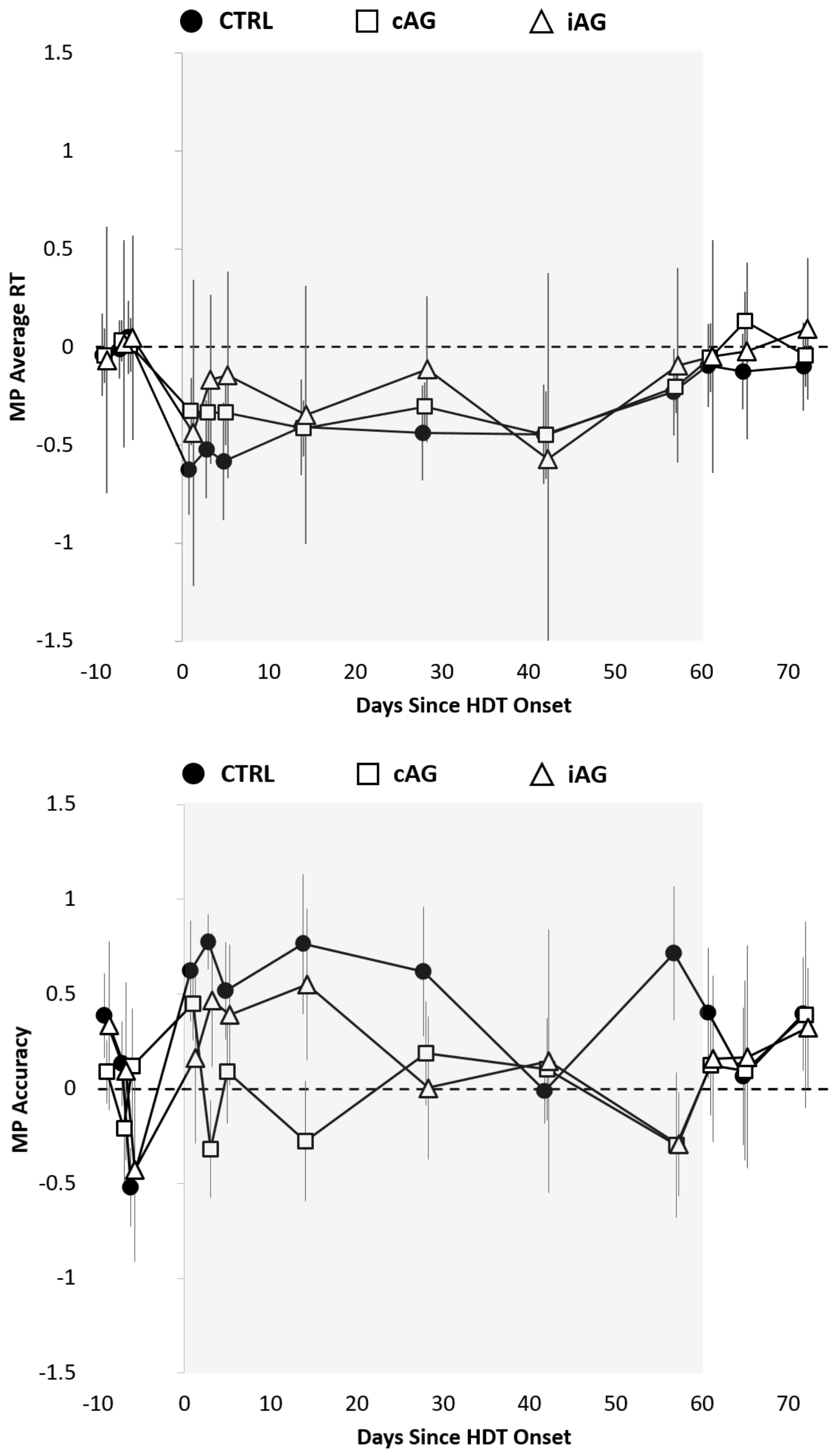

Figure S2: Speed and accuracy on the Motor Praxis (MP) test relative to the 60-day head-down tilt (HDT) bed rest period (gray background) for the control group (black circles), continuous artificial gravity group (cAG; white squares) and intermittent artificial gravity group (iAG, white triangles). Estimates reflect unadjusted means (standard errors) z-transformed based on baseline (pre-HDT) performance. To reflect the analytical approach (adjusting for baseline performance), means were shifted within groups to reflect a pre-HDT baseline performance of 0 (zero).

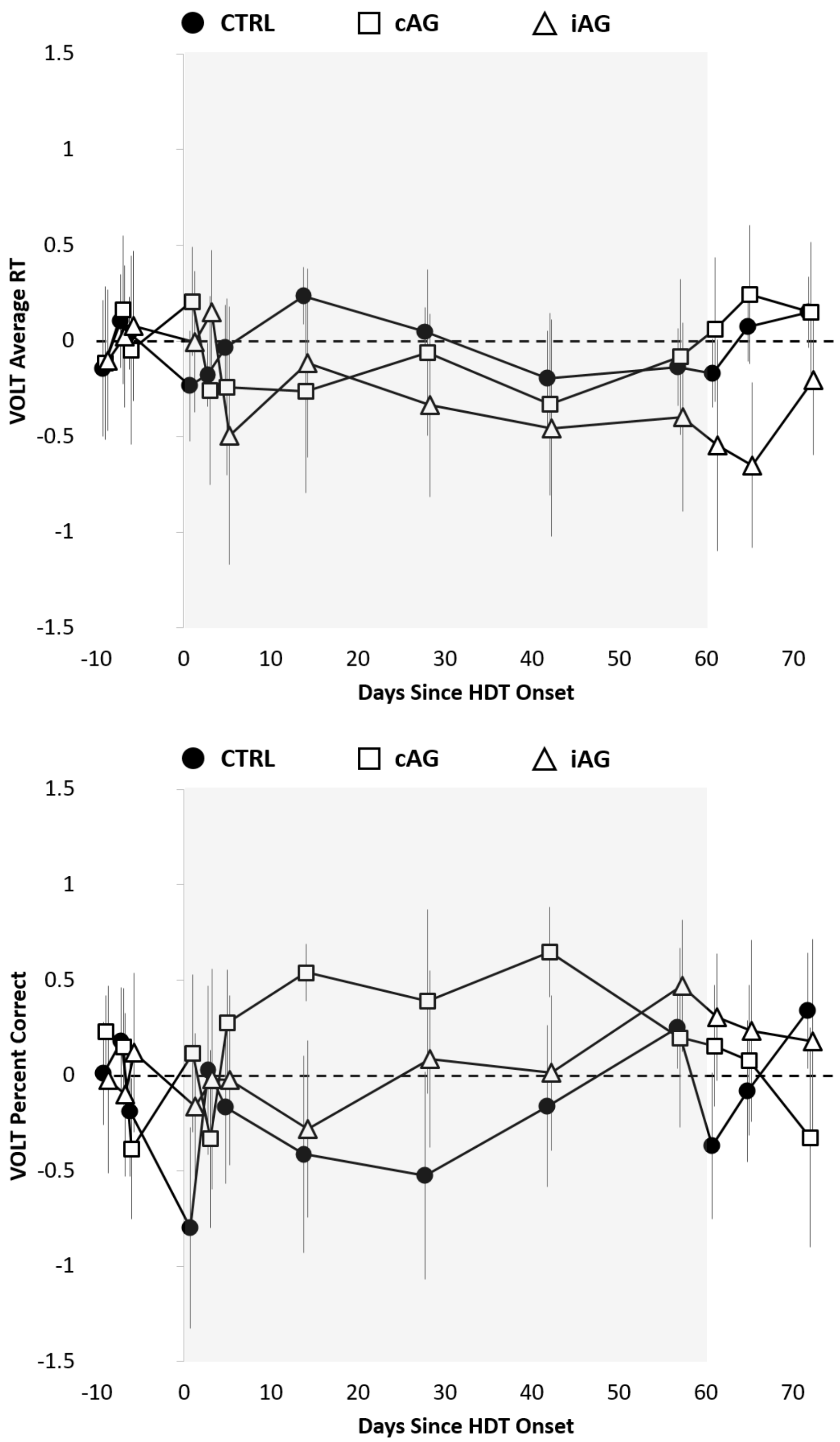

Figure S3: Speed and accuracy on the Visual Object learning Test (VOLT) relative to the 60-day head-down tilt (HDT) bed rest period (gray background) for the control group (black circles), continuous artificial gravity group (cAG; white squares) and intermittent artificial gravity group (iAG, white triangles). Estimates reflect unadjusted means (standard errors) z-transformed based on baseline (pre-HDT) performance. To reflect the analytical approach (adjusting for baseline performance), means were shifted within groups to reflect a pre-HDT baseline performance of 0 (zero).

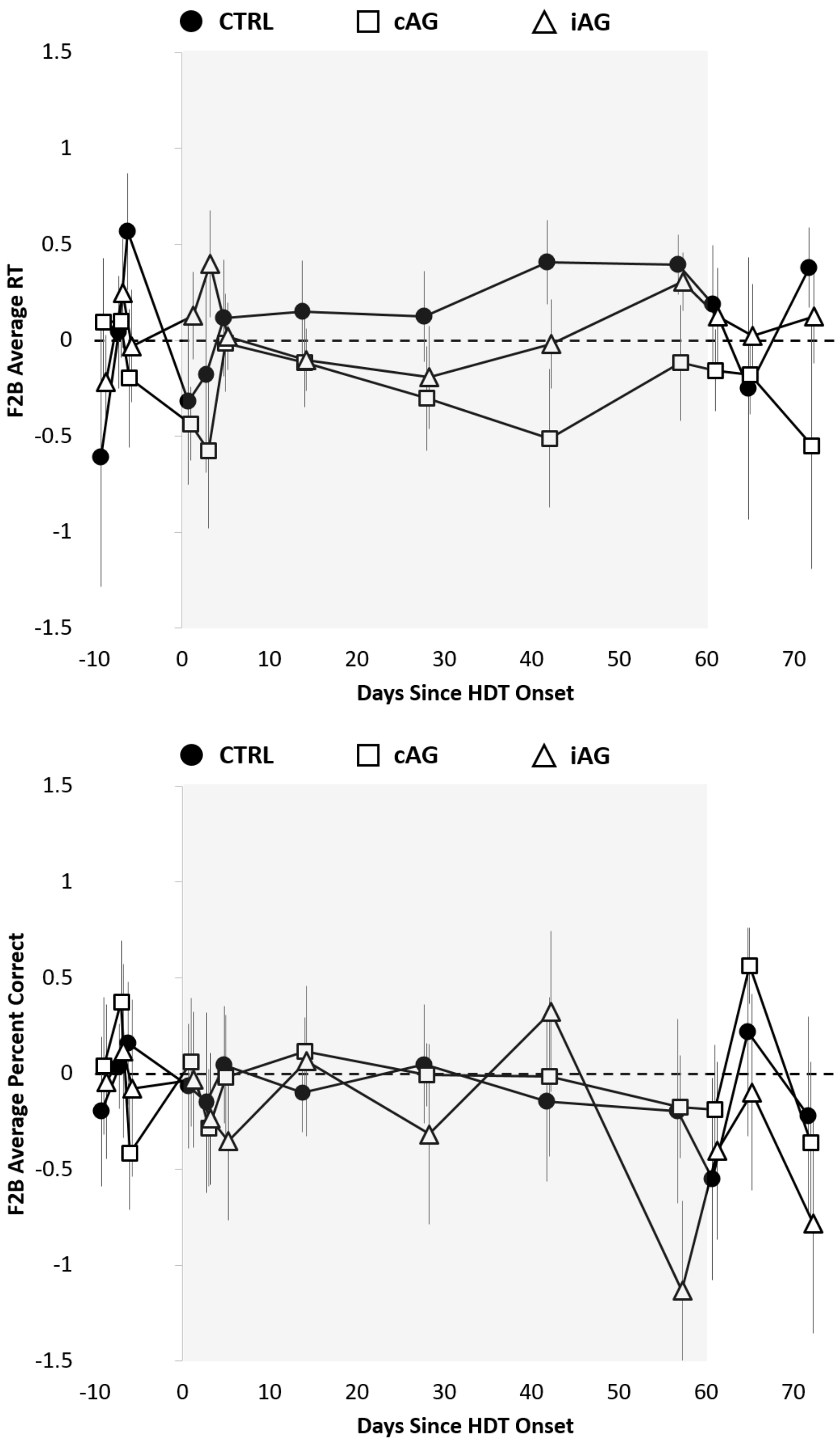

Figure S4: Speed and accuracy on the Fractal 2-Back (F2B) test relative to the 60-day head-down tilt (HDT) bed rest period (gray background) for the control group (black circles), continuous artificial gravity group (cAG; white squares) and intermittent artificial gravity group (iAG, white triangles). Estimates reflect unadjusted means (standard errors) z-transformed based on baseline (pre-HDT) performance. To reflect the analytical approach (adjusting for baseline performance), means were shifted within groups to reflect a pre-HDT baseline performance of 0 (zero).

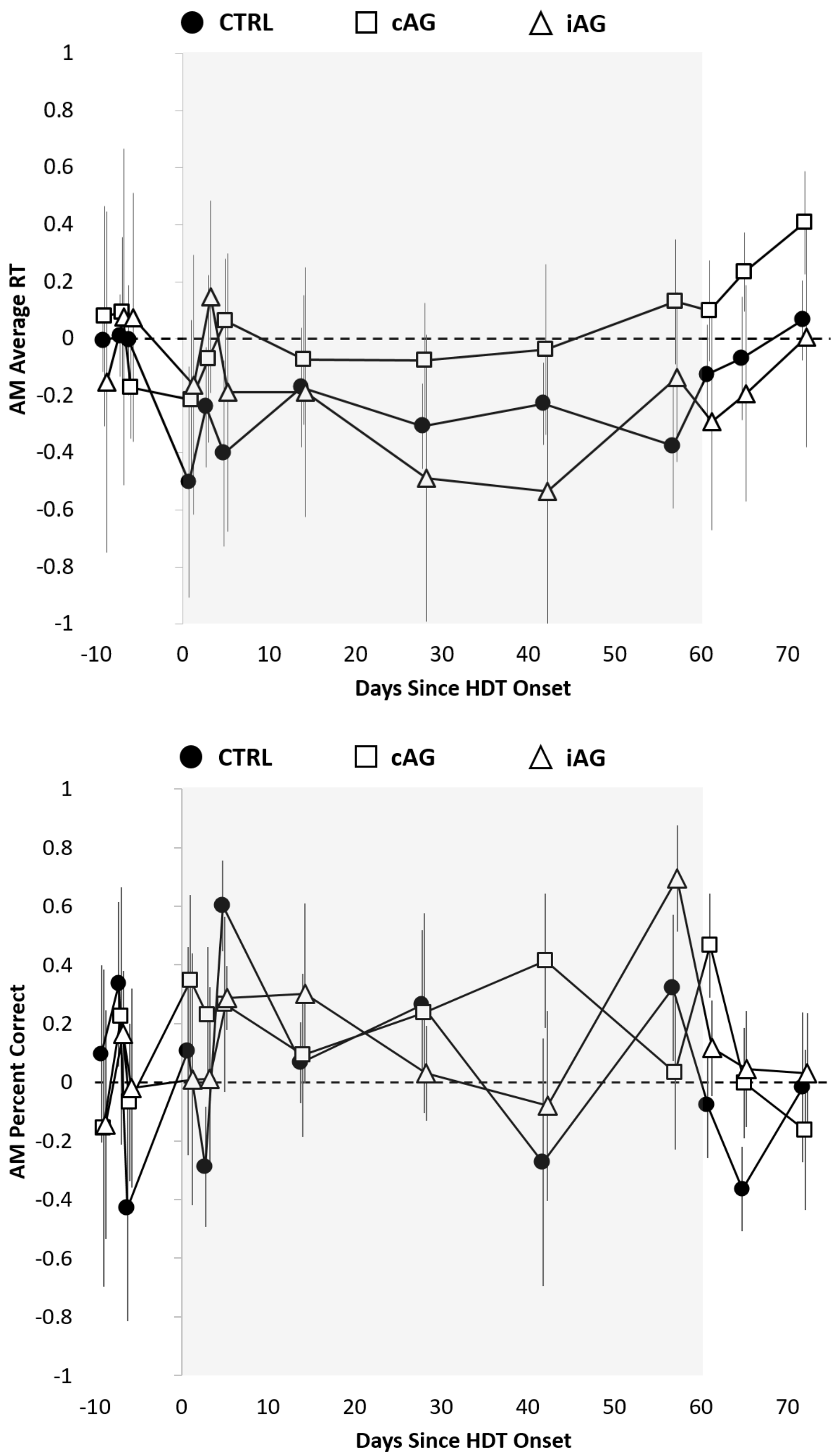

Figure S5: Speed and accuracy on the Abstract Matching (AM) test relative to the 60-day head-down tilt (HDT) bed rest period (gray background) for the control group (black circles), continuous artificial gravity group (cAG; white squares) and intermittent artificial gravity group (iAG, white triangles). Estimates reflect unadjusted means (standard errors) z-transformed based on baseline (pre-HDT) performance. To reflect the analytical approach (adjusting for baseline performance), means were shifted within groups to reflect a pre-HDT baseline performance of 0 (zero).

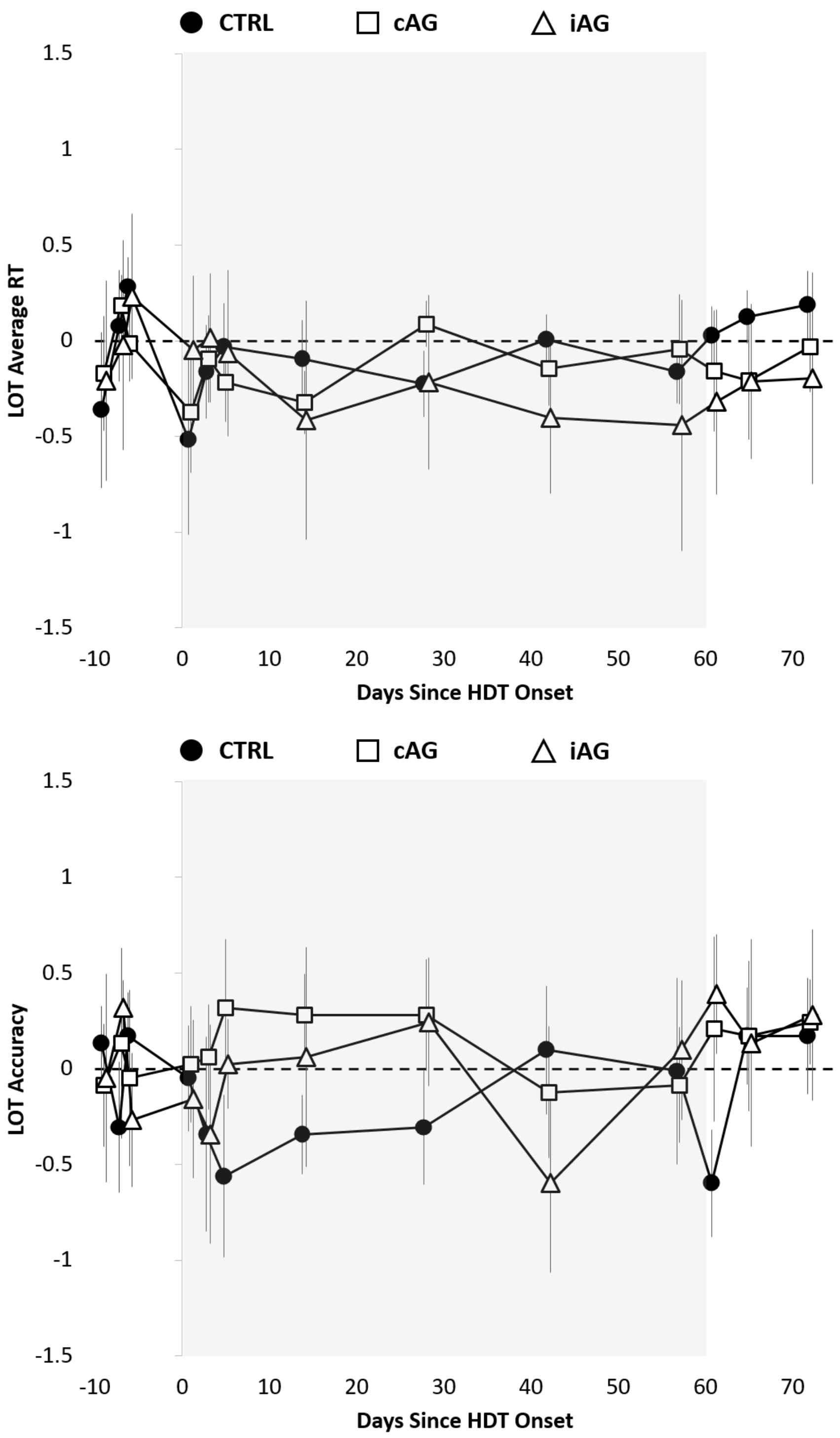

Figure S6: Speed and accuracy on the Line Orientation Test (LOT) relative to the 60-day head-down tilt (HDT) bed rest period (gray background) for the control group (black circles), continuous artificial gravity group (cAG; white squares) and intermittent artificial gravity group (iAG, white triangles). Estimates reflect unadjusted means (standard errors) z-transformed based on baseline (pre-HDT) performance. To reflect the analytical approach (adjusting for baseline performance), means were shifted within groups to reflect a pre-HDT baseline performance of 0 (zero).

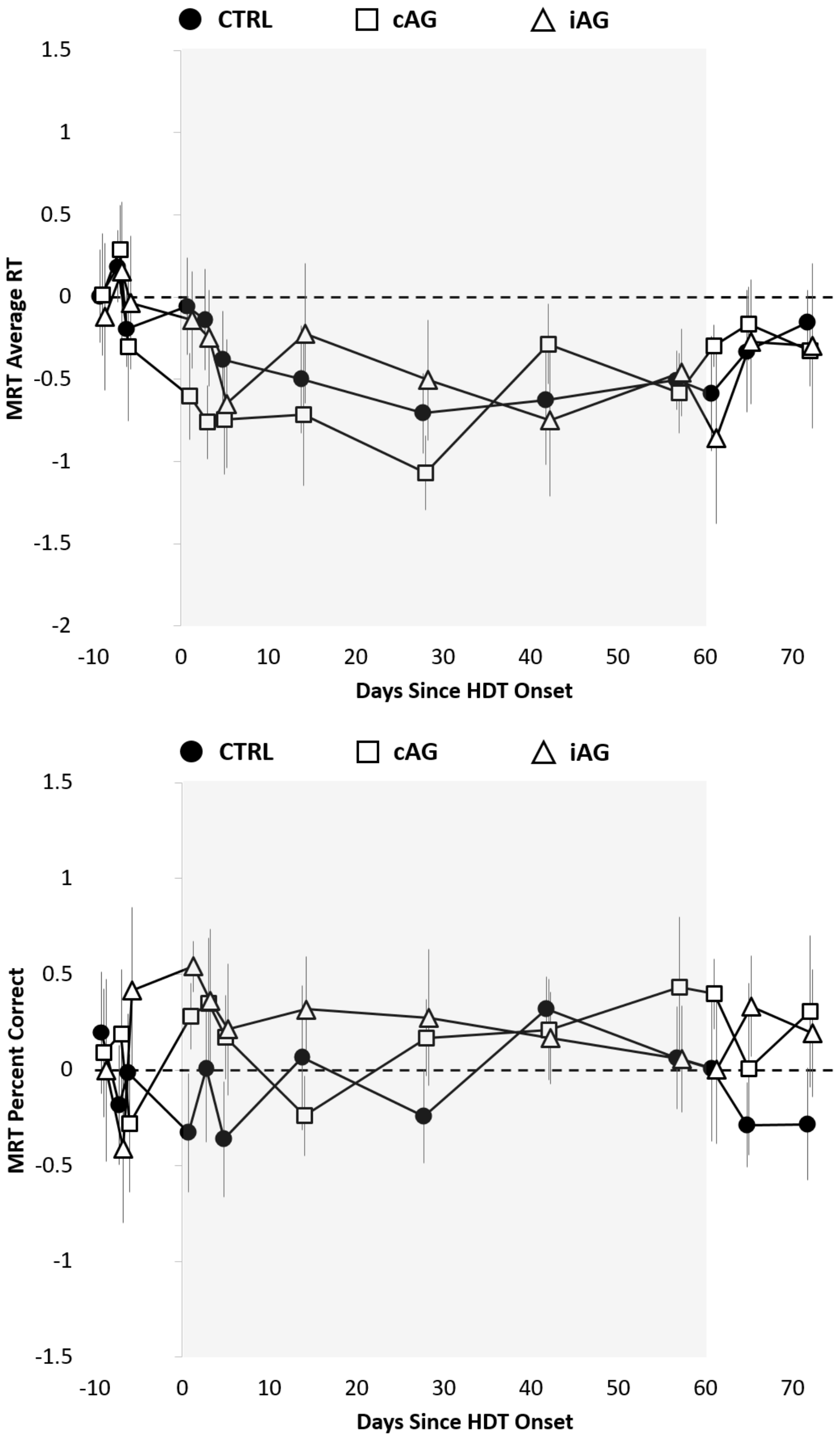

Figure S7: Speed and accuracy on the Matrix Reasoning Test (MRT) relative to the 60-day head-down tilt (HDT) bed rest period (gray background) for the control group (black circles), continuous artificial gravity group (cAG; white squares) and intermittent artificial gravity group (iAG, white triangles). Estimates reflect unadjusted means (standard errors) z-transformed based on baseline (pre-HDT) performance. To reflect the analytical approach (adjusting for baseline performance), means were shifted within groups to reflect a pre-HDT baseline performance of 0 (zero).

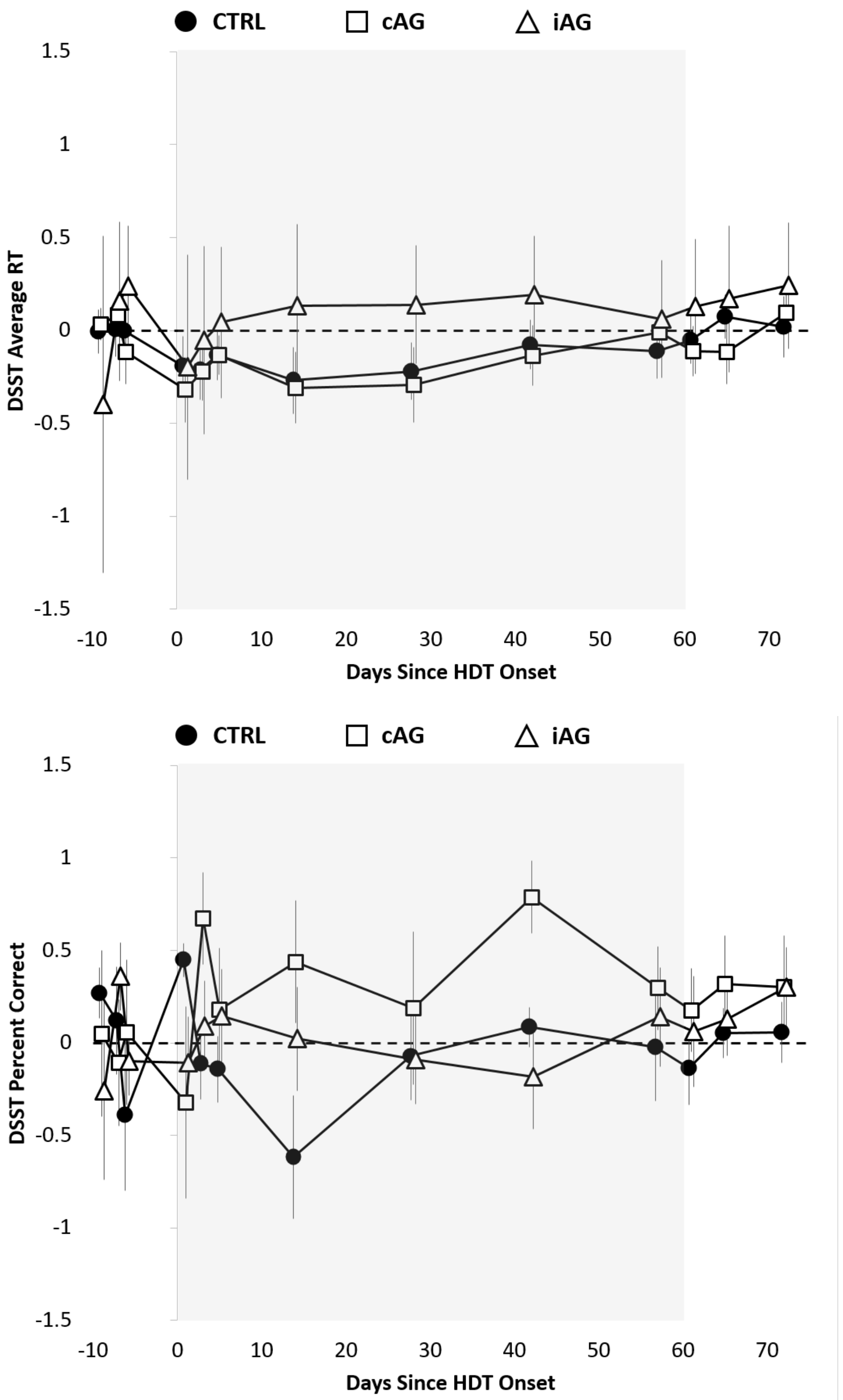

Figure S8: Speed and accuracy on the Digit Symbol Substitution Test (DSST) relative to the 60-day head-down tilt (HDT) bed rest period (gray background) for the control group (black circles), continuous artificial gravity group (cAG; white squares) and intermittent artificial gravity group (iAG, white triangles). Estimates reflect unadjusted means (standard errors) z-transformed based on baseline (pre-HDT) performance. To reflect the analytical approach (adjusting for baseline performance), means were shifted within groups to reflect a pre-HDT baseline performance of 0 (zero).

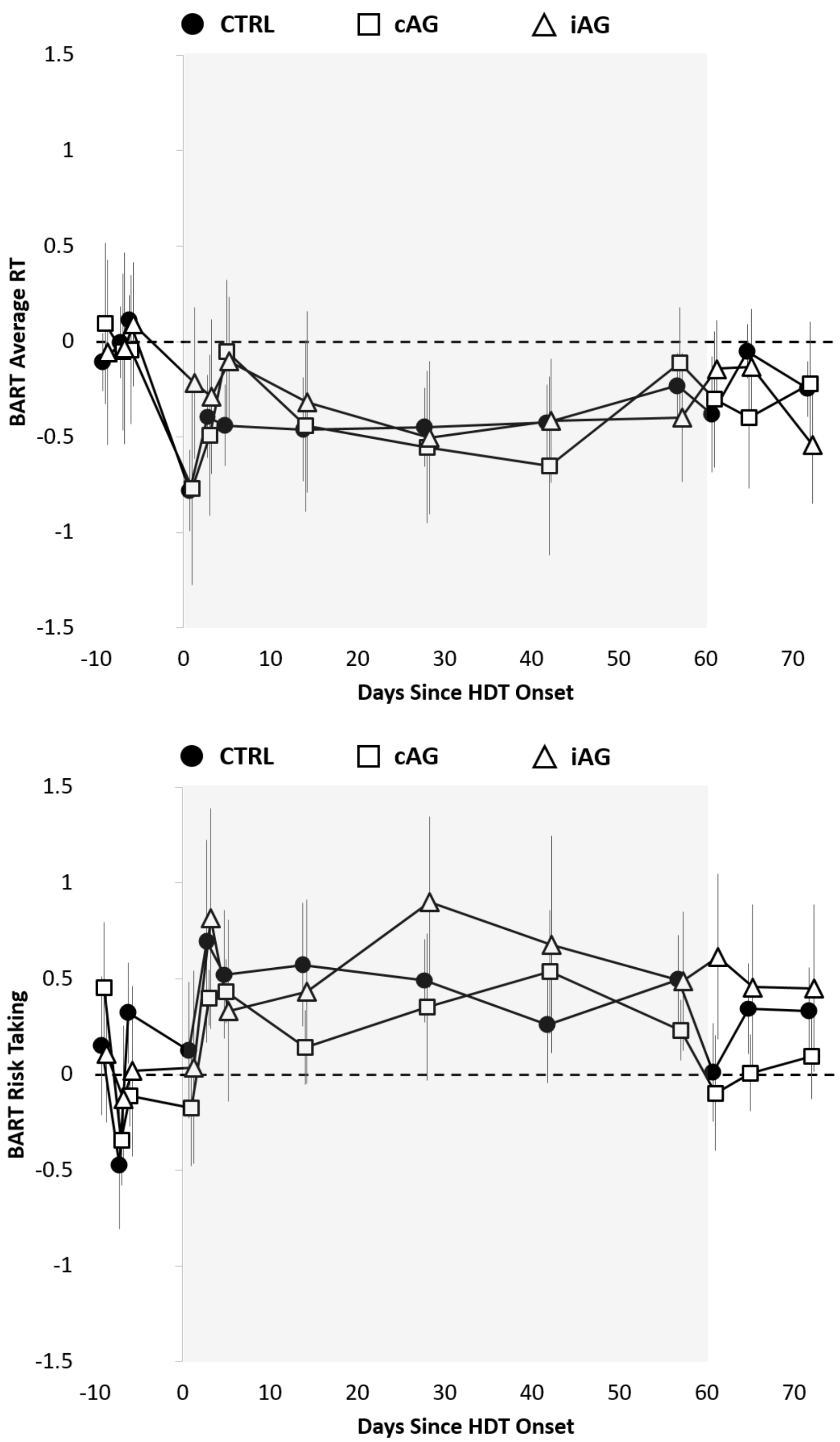

Figure S9: Speed and accuracy on the Balloon Analog Risk Test (BART) relative to the 60-day head-down tilt (HDT) bed rest period (gray background) for the control group (black circles), continuous artificial gravity group (cAG; white squares) and intermittent artificial gravity group (iAG, white triangles). Estimates reflect unadjusted means (standard errors) z-transformed based on baseline (pre-HDT) performance. To reflect the analytical approach (adjusting for baseline performance), means were shifted within groups to reflect a pre-HDT baseline performance of 0 (zero).

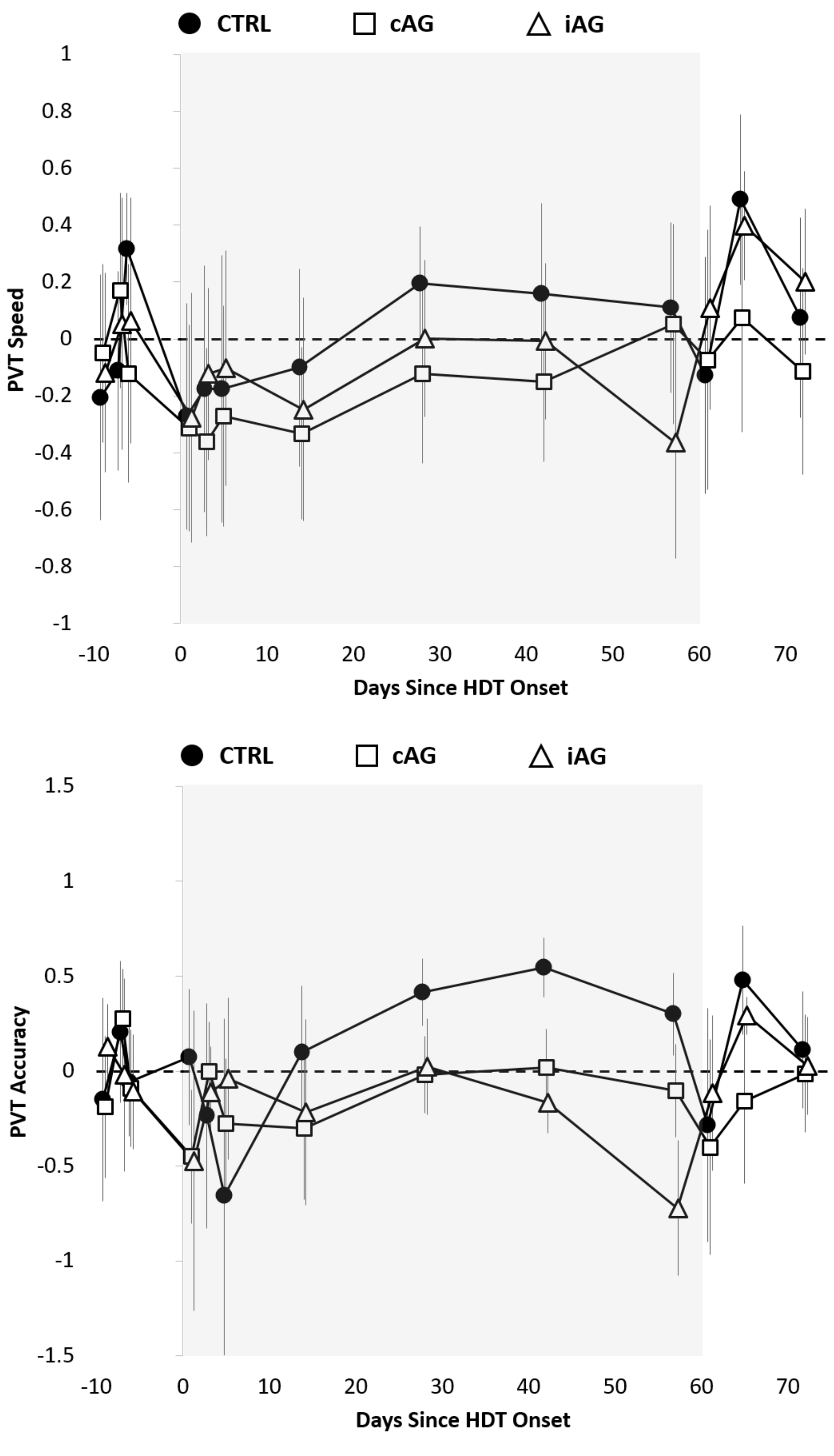

Figure S10: Speed and accuracy on the Psychomotor Vigilance Test (PVT) relative to the 60-day head-down tilt (HDT) bed rest period (gray background) for the control group (black circles), continuous artificial gravity group (cAG; white squares) and intermittent artificial gravity group (iAG, white triangles). Estimates reflect unadjusted means (standard errors) z-transformed based on baseline (pre-HDT) performance. To reflect the analytical approach (adjusting for baseline performance), means were shifted within groups to reflect a pre-HDT baseline performance of 0 (zero).

Table S1: Comparison of baseline assessments

	Baseline Performance L-9/-7/-6				
**Variable**	**Control**	**Continuous AG**	**Intermittent AG**	**Test III**	**Adj. Test III**
MP Average RT [ms]	892.0 (725.5; 1058.5)	835.7 (669.2; 1058.5)	991.0 (824.5; 1058.5)	0.3973	0.3517
VOLT Average RT [ms]	1491.6 (1081.7; 1901.4)	1759.4 (1349.5; 1901.4)	1597.9 (1188.0; 1901.4)	0.6325	0.4726
F2B Average RT [ms]	621.7 (540.8; 702.5)	575.3 (494.9; 702.5)	590.7 (510.4; 702.5)	0.6944	0.7091
AM Average RT [ms]	1737.8 (1081.4; 2394.1)	2128.7 (1472.4; 2394.1)	1834.9 (1178.6; 2394.1)	0.6650	0.4149
LOT Average RT [ms]	5093.6 (3752.7; 6434.6)	5117.7 (3776.7; 6434.6)	5719.5 (4378.6; 6434.6)	0.7421	0.7814
ERT Average RT [ms]	1495.6 (1120.0; 1871.3)	1529.7 (1154.1; 1871.3)	1747.9 (1372.2; 1871.3)	0.5719	0.6172
MRT Average RT [ms]	6520.0 (4425.6; 8614.4)	8446.8 (6352.4; 8614.4)	8547.3 (6447.7; 8614.4)	0.2973	0.2073
DSST Average RT [ms]	1332.3 (965.9; 1698.6)	1296.5 (930.1; 1698.6)	1471.3 (1104.9; 1698.6)	0.7625	0.8162
BART Average RT [ms]	1286.7 (738.9; 1834.5)	1742.6 (1194.8; 1834.5)	1307.9 (760.0; 1834.5)	0.4012	0.1950
PVT Slowness [10 - 1/s]	5.40 (5.00; 5.80)	5.33 (4.92; 5.80)	5.23 (4.83; 5.80)	0.8125	0.4667
MP Accuracy [%]	25.4 (19.0; 31.7)	28.0 (21.7; 31.7)	33.5 (27.2; 31.7)	0.1813	0.1918
VOLT Percent Correct	91.7 (86.0; 97.3)	87.1 (81.5; 97.3)	86.4 (80.8; 97.3)	0.3509	0.3055
F2B Average Percent Correct	85.0 (78.5; 91.6)	87.1 (80.6; 91.6)	85.6 (79.1; 91.6)	0.8917	0.9872
AM Percent Correct	78.7 (72.1; 85.3)	77.4 (70.8; 85.3)	80.6 (74.0; 85.3)	0.7761	0.7792
LOT Accuracy [%]	78.2 (72.5; 84.0)	76.5 (70.7; 84.0)	76.5 (70.7; 84.0)	0.8781	0.9507
ERT Percent Correct	68.9 (62.4; 75.3)	70.8 (64.3; 75.3)	73.7 (67.2; 75.3)	0.5481	0.4748
MRT Percent Correct	73.4 (65.3; 81.4)	77.1 (69.1; 81.4)	75.4 (67.3; 81.4)	0.7871	0.8370
DSST Percent Correct	98.4 (96.7; 100.0)	95.9 (94.2; 100.0)	97.7 (96.0; 100.0)	0.0917	**0.0495**
BART Risk Score P [%]	65.9 (59.3; 72.5)	71.1 (64.5; 72.5)	60.4 (53.8; 72.5)	0.0816	0.0708
PVT Accuracy [%]	92.1 (87.6; 96.5)	94.4 (90.0; 96.5)	94.2 (89.7; 96.5)	0.6988	0.6365
Speed	0.11 (-0.40; 0.62)	-0.03 (-0.54; 0.62)	-0.08 (-0.59; 0.62)	0.8549	0.7974
Accuracy	-0.02 (-0.31; 0.28)	-0.06 (-0.36; 0.28)	0.08 (-0.21; 0.28)	0.7687	0.4830
Efficiency	0.05 (-0.30; 0.40)	-0.05 (-0.40; 0.40)	0.00 (-0.35; 0.40)	0.9270	0.7005
Sleep Duration [h]	7.54 (7.31; 7.76)	7.61 (7.39; 7.76)	7.55 (7.33; 7.76)	0.8756	0.7553
Poor Sleep Quality	4.6 (3.4; 5.8)	3.9 (2.7; 5.8)	5.4 (4.2; 5.8)	0.2209	0.1980
Low Workload	5.0 (3.9; 6.1)	4.0 (3.0; 6.1)	3.9 (2.8; 6.1)	0.2934	0.3350
Sleepy	4.4 (3.2; 5.6)	5.0 (3.8; 5.6)	5.1 (3.9; 5.6)	0.6608	0.7496
Unhappy	2.7 (1.6; 3.7)	2.9 (1.8; 3.7)	3.0 (2.0; 3.7)	0.8700	0.8887
Healthy	8.5 (7.1; 9.8)	8.5 (7.2; 9.8)	7.4 (6.1; 9.8)	0.3811	0.3829
Physically Exhausted	3.5 (2.2; 4.7)	4.7 (3.4; 4.7)	4.8 (3.6; 4.7)	0.2171	0.2240
Mentally Fatigued	4.0 (2.5; 5.4)	4.0 (2.5; 5.4)	4.3 (2.8; 5.4)	0.9516	0.9626
Stressed	2.2 (1.0; 3.5)	2.2 (0.9; 3.5)	4.0 (2.7; 3.5)	0.0777	0.0803
Fresh	5.0 (3.7; 6.2)	5.1 (3.8; 6.2)	4.2 (2.9; 6.2)	0.5556	0.5976
Not Depressed	9.3 (8.2; 10.3)	8.9 (7.9; 10.3)	8.9 (7.9; 10.3)	0.8414	0.8505
Not Bored	8.3 (6.9; 9.8)	8.3 (6.8; 9.8)	7.7 (6.2; 9.8)	0.7611	0.5699
Lonely	1.5 (0.0; 3.0)	1.5 (0.1; 3.0)	1.6 (0.2; 3.0)	0.9918	0.9578
Not Monotonous	7.8 (6.2; 9.5)	7.4 (5.8; 9.5)	7.8 (6.2; 9.5)	0.9172	0.8925
Age (years ±SD)	34.3 (7.9)	31.9 (9.7)	33.8 (10.8)	0.872	N/A
Male N (%)	6 (75%)	5 (62.5%)	5 (62.5%)	0.829	N/A

Mixed effect model estimates (shown with 95% confidence limits) are based on data of test bouts performed 9, 7 and 6 days prior to initiation of the head-down tilt bed rest period. Adjusted p-values reflect models adjusted for age and sex. Estimates for self-report data reflect points on an 11-point scale (variables are listed by anchors for high values). For Age, values in parenthesis reflect standard deviation. For Male, the p-value is based on a χ^2 test. MP: Motor Praxis; VOLT: Visual Object Learning Test; F2B: Fractal 2-Back; AM: Abstract Matching; LOT: Line Orientation Test; ERT: Emotion Recognition Test; MRT: Matrix Reasoning Test; DSST: Digit Symbol Substitution Test; BART: Balloon Analog Risk Test; PVT: Psychomotor Vigilance Test; ms: milliseconds; AG: artificial gravity; Adj.: adjusted

Table S2: Mixed effect model results for the head-down tilt bed rest period

	Main Effects						Contrasts						
**Variable**	**CTRL**	**cAG**	**iAG**	**p(CTRL)**	**p(cAG)**	**p(iAG)**	**cAG-CTRL**	**iAG-CTRL**	**cAG-iAG**	**p(cAG-CTRL)**	**p(iAG-CTRL)**	**p(cAG-iAG)**	**Test III**
MP Speed	-0.48 (-0.64; -0.31)	-0.35 (-0.52; -0.18)	-0.24 (-0.41; -0.07)	<.0001***	0.0004**	0.0073*	0.13 (-0.11; 0.36)	0.23 (-0.01; 0.47)	-0.11 (-0.35; 0.14)	0.2717	0.0551	0.3729	0.1487
VOLT Speed	-0.03 (-0.32; 0.26)	-0.21 (-0.50; 0.08)	-0.21 (-0.50; 0.07)	0.8172	0.1503	0.1368	-0.18 (-0.60; 0.25)	-0.18 (-0.59; 0.23)	0.00 (-0.41; 0.41)	0.3926	0.3688	0.9895	0.5951
F2B Speed	0.09 (-0.14; 0.32)	-0.27 (-0.50; -0.04)	0.08 (-0.14; 0.31)	0.4448	0.0232	0.4521	-0.36 (-0.69; -0.03)	0.00 (-0.33; 0.32)	-0.35 (-0.67; -0.03)	0.0357	0.9856	0.0330	0.0521
AM Speed	-0.22 (-0.52; 0.08)	-0.17 (-0.47; 0.14)	-0.19 (-0.49; 0.10)	0.1380	0.2661	0.1837	0.05 (-0.38; 0.49)	0.03 (-0.39; 0.44)	0.03 (-0.40; 0.45)	0.7944	0.8928	0.8927	0.9657
LOT Speed	-0.15 (-0.45; 0.15)	-0.15 (-0.44; 0.15)	-0.26 (-0.55; 0.04)	0.3077	0.3152	0.0844	0.00 (-0.42; 0.42)	-0.11 (-0.53; 0.31)	0.11 (-0.31; 0.53)	0.9883	0.5929	0.5805	0.8172
ERT Speed	-0.59 (-1.05; -0.13)	-0.45 (-0.90; 0.01)	-0.66 (-1.12; -0.20)	0.0152	0.0549	0.0074*	0.14 (-0.51; 0.79)	-0.08 (-0.73; 0.58)	0.22 (-0.44; 0.87)	0.6553	0.8123	0.4962	0.7815
MRT Speed	-0.27 (-0.55; 0.01)	-0.75 (-1.02; -0.49)	-0.51 (-0.78; -0.25)	0.0593	<.0001	0.0007**	-0.48 (-0.88; -0.09)	-0.24 (-0.64; 0.15)	-0.24 (-0.61; 0.13)	0.0200	0.2096	0.1901	0.0614
DSST Speed	-0.14 (-0.30; 0.01)	-0.18 (-0.34; -0.03)	-0.01 (-0.16; 0.15)	0.0700	0.0252	0.9298	-0.04 (-0.26; 0.18)	0.14 (-0.09; 0.36)	-0.17 (-0.40; 0.05)	0.7213	0.2123	0.1153	0.2468
BART Speed	-0.39 (-0.71; -0.07)	-0.56 (-0.89; -0.22)	-0.26 (-0.59; 0.06)	0.0199	0.0026*	0.1034	-0.17 (-0.65; 0.31)	0.13 (-0.32; 0.58)	-0.29 (-0.78; 0.19)	0.4693	0.5626	0.2162	0.4555
PVT Speed	-0.02 (-0.31; 0.26)	-0.21 (-0.49; 0.06)	-0.17 (-0.45; 0.11)	0.8547	0.1205	0.2152	-0.19 (-0.59; 0.21)	-0.15 (-0.55; 0.26)	-0.04 (-0.44; 0.35)	0.3297	0.4581	0.8233	0.5916
MP Accuracy	0.44 (0.13; 0.76)	-0.04 (-0.35; 0.26)	0.37 (0.05; 0.69)	0.0087	0.7678	0.0273	-0.49 (-0.92; -0.05)	-0.07 (-0.54; 0.40)	-0.41 (-0.87; 0.04)	0.0314	0.7467	0.0716	0.0648
VOLT Accuracy	-0.21 (-0.62; 0.20)	0.25 (-0.14; 0.65)	-0.04 (-0.43; 0.36)	0.2964	0.1966	0.8481	0.46 (-0.12; 1.05)	0.17 (-0.41; 0.75)	0.29 (-0.26; 0.84)	0.1140	0.5410	0.2865	0.2627
F2B Accuracy	-0.09 (-0.43; 0.24)	-0.03 (-0.36; 0.31)	-0.24 (-0.57; 0.09)	0.5595	0.8749	0.1404	0.07 (-0.40; 0.54)	-0.15 (-0.62; 0.32)	0.22 (-0.25; 0.69)	0.7630	0.5149	0.3426	0.6161
AM Accuracy	0.09 (-0.19; 0.37)	0.15 (-0.14; 0.43)	0.29 (0.01; 0.57)	0.5071	0.2946	0.0460	0.05 (-0.35; 0.46)	0.20 (-0.20; 0.60)	-0.14 (-0.54; 0.26)	0.7790	0.3146	0.4646	0.5754
LOT Accuracy	-0.15 (-0.66; 0.36)	0.07 (-0.44; 0.58)	-0.12 (-0.63; 0.38)	0.5394	0.7671	0.6127	0.23 (-0.50; 0.95)	0.03 (-0.70; 0.75)	0.20 (-0.52; 0.92)	0.5232	0.9364	0.5712	0.7785
ERT Accuracy	0.12 (-0.28; 0.53)	-0.01 (-0.41; 0.39)	0.09 (-0.31; 0.50)	0.5222	0.9775	0.6413	-0.13 (-0.69; 0.44)	-0.03 (-0.61; 0.55)	-0.10 (-0.67; 0.48)	0.6350	0.9057	0.7280	0.8826
MRT Accuracy	-0.17 (-0.47; 0.12)	0.27 (-0.01; 0.56)	0.30 (0.02; 0.59)	0.2259	0.0607	0.0393	0.45 (0.03; 0.86)	0.48 (0.07; 0.89)	-0.03 (-0.44; 0.38)	0.0351	0.0248	0.8842	0.0449
DSST Accuracy	0.31 (0.02; 0.61)	-0.15 (-0.46; 0.16)	0.10 (-0.18; 0.38)	0.0389	0.3216	0.4637	-0.46 (-0.92; 0.00)	-0.21 (-0.61; 0.18)	-0.25 (-0.67; 0.18)	0.0483	0.2743	0.2364	0.1348
BART Risk Taking	0.41 (-0.15; 0.96)	0.48 (-0.11; 1.08)	0.36 (-0.23; 0.96)	0.1402	0.1059	0.2161	0.07 (-0.74; 0.89)	-0.04 (-0.86; 0.77)	0.12 (-0.78; 1.02)	0.8502	0.9120	0.7857	0.9616
PVT Accuracy	0.07 (-0.15; 0.30)	-0.17 (-0.39; 0.05)	-0.23 (-0.45; -0.01)	0.5255	0.1289	0.0414	-0.24 (-0.56; 0.08)	-0.30 (-0.62; 0.02)	0.06 (-0.25; 0.37)	0.1343	0.0625	0.7101	0.1459
Speed	-0.23 (-0.35; -0.11)	-0.31 (-0.43; -0.19)	-0.25 (-0.37; -0.13)	0.0009*	<.0001***	0.0004**	-0.08 (-0.25; 0.09)	-0.02 (-0.19; 0.15)	-0.06 (-0.23; 0.11)	0.3476	0.8201	0.4634	0.6073
Accuracy	0.03 (-0.12; 0.17)	0.09 (-0.06; 0.24)	0.02 (-0.12; 0.17)	0.7189	0.2261	0.7321	0.06 (-0.15; 0.28)	0.00 (-0.21; 0.21)	0.06 (-0.15; 0.28)	0.5339	0.9926	0.5357	0.7710
Efficiency	-0.10 (-0.20; -0.01)	-0.11 (-0.20; -0.01)	-0.12 (-0.21; -0.02)	0.0328	0.0280	0.0176	0.00 (-0.14; 0.13)	-0.01 (-0.14; 0.12)	0.01 (-0.12; 0.14)	0.9519	0.8478	0.8970	0.9803
Sleep Duration [h]	-0.18 (-0.47; 0.10)	-0.06 (-0.34; 0.23)	-0.09 (-0.38; 0.19)	0.2131	0.6917	0.5151	0.25 (-0.19; 0.69)	0.13 (-0.30; 0.57)	0.12 (-0.32; 0.56)	0.2495	0.5350	0.5702	0.5055
Poor Sleep Quality	-0.4 (-1.3; 0.6)	0.5 (-0.4; 1.5)	-0.8 (-1.8; 0.1)	0.4456	0.2761	0.0885	0.7 (-1.3; 2.6)	0.0 (-1.9; 1.9)	0.7 (-1.3; 2.7)	0.4764	0.9688	0.4746	0.7130
Low Workload	2.0 (1.1; 2.9)	1.0 (0.1; 1.8)	0.9 (0.1; 1.8)	<.0001****	0.0229*	0.0346	-1.6 (-2.5; -0.6)	-1.7 (-2.7; -0.8)	0.2 (-0.7; 1.0)	0.0023*	0.0010*	0.7040	0.0019*
Sleepy	0.5 (-0.3; 1.4)	0.2 (-0.6; 1.1)	0.3 (-0.6; 1.1)	0.2075	0.5803	0.5235	-0.1 (-1.5; 1.4)	0.1 (-1.4; 1.6)	-0.2 (-1.6; 1.3)	0.9293	0.8678	0.7957	0.9651
Unhappy	0.7 (-0.1; 1.5)	0.5 (-0.3; 1.3)	1.2 (0.4; 2.0)	0.0822	0.2361	0.0048*	-0.3 (-1.7; 1.1)	0.5 (-0.9; 1.9)	-0.8 (-2.2; 0.6)	0.6371	0.4930	0.2507	0.5038
Healthy	-2.0 (-3.0; -1.1)	-0.9 (-1.9; 0.0)	-1.8 (-2.8; -0.9)	<.0001***	0.0548	0.0002**	1.4 (-0.7; 3.5)	-0.2 (-2.3; 1.9)	1.6 (-0.5; 3.8)	0.1711	0.8419	0.1306	0.2421
Physically Exhausted	0.9 (0.0; 1.8)	-0.3 (-1.2; 0.6)	0.6 (-0.2; 1.5)	0.0404	0.4959	0.1540	-0.8 (-2.5; 0.9)	0.3 (-1.4; 2.0)	-1.1 (-2.7; 0.5)	0.3203	0.7448	0.1672	0.3488
Mentally Fatigued	0.4 (-0.5; 1.2)	1.0 (0.2; 1.9)	0.7 (-0.2; 1.5)	0.3757	0.0184*	0.1250	0.6 (-1.1; 2.3)	0.4 (-1.3; 2.0)	0.2 (-1.4; 1.9)	0.4634	0.6448	0.7792	0.7545
Stressed	0.5 (-0.4; 1.3)	1.3 (0.4; 2.2)	0.0 (-0.9; 0.8)	0.3095	0.0038*	0.9147	0.6 (-1.4; 2.6)	0.2 (-2.1; 2.4)	0.4 (-1.8; 2.6)	0.5529	0.8628	0.7109	0.8284
Fresh	0.0 (-0.8; 0.9)	-0.4 (-1.3; 0.4)	0.0 (-0.9; 0.8)	0.9241	0.3340	0.9457	-0.4 (-2.1; 1.3)	-0.4 (-2.1; 1.3)	0.0 (-1.7; 1.7)	0.6207	0.6088	0.9843	0.8396
Not Depressed	-0.5 (-1.3; 0.3)	-1.1 (-1.8; -0.3)	-1.5 (-2.3; -0.7)	0.2460	0.0084*	0.0001**	-0.5 (-2.1; 1.2)	-1.2 (-2.8; 0.5)	0.7 (-1.0; 2.3)	0.5457	0.1548	0.3920	0.3491
Not Bored	0.1 (-0.7; 0.8)	-1.3 (-2.0; -0.5)	-0.7 (-1.5; 0.0)	0.8362	0.0009**	0.0551	-1.3 (-2.3; -0.2)	-1.1 (-2.2; 0.0)	-0.2 (-1.3; 0.9)	0.0246	0.0523	0.7425	0.0520
Lonely	0.5 (-0.3; 1.3)	1.6 (0.8; 2.5)	1.2 (0.3; 2.0)	0.2519	0.0001**	0.0061*	0.9 (-0.6; 2.3)	0.7 (-0.8; 2.1)	0.2 (-1.3; 1.7)	0.2428	0.3642	0.7797	0.4651
Not Monotonous	-2.3 (-3.1; -1.4)	-1.3 (-2.1; -0.5)	-1.4 (-2.2; -0.5)	<.0001****	0.0022*	0.0015**	0.9 (-0.3; 2.1)	1.0 (-0.2; 2.2)	0.0 (-1.3; 1.2)	0.1301	0.1093	0.9355	0.1973

All models were adjusted for sex and age. Models with cognitive outcomes were additionally adjusted for baseline performance. Estimates for cognitive tests reflect z-scores. As z-transformation was based on baseline performance, an estimate of 0 (zero) reflects baseline performance. Estimates for self-report data reflect points on an 11-point scale (variables are listed by anchors for high values). As sleep and subjective outcomes were not z-transformed, estimates for these variables were not adjusted for baseline values but are based on a direct contrast between the head-down tilt and baseline period instead. Est.: Estimate; CI: Confidence Interval; CTRL: Control; cAG: continuous Artificial Gravity; iAG: intermittent Artificial Gravity; *adjusted p<0.05; **adjusted p<0.01; ***adjusted p<0.001; ****adjusted p<0.0001; MP: Motor Praxis; VOLT: Visual Object Learning Test; F2B: Fractal 2-Back; AM: Abstract Matching; LOT: Line Orientation Test; ERT: Emotion Recognition Test; MRT: Matrix Reasoning Test; DSST: Digit Symbol Substitution Test; BART: Balloon Analog Risk Test; PVT: Psychomotor Vigilance Test

Table S3: Mixed effect model results for the recovery period

	Main Effects						Contrasts						
Variable	CTRL	cAG	iAG	p(CTRL)	p(cAG)	p(iAG)	cAG-CTRL	iAG-CTRL	cAG-iAG	p(cAG-CTRL)	p(iAG-CTRL)	p(cAG-iAG)	Test III
MP Speed	-0.11 (-0.28; 0.06)	0.08 (-0.09; 0.25)	-0.06 (-0.23; 0.11)	0.1984	0.3402	0.4881	0.19 (-0.05; 0.43)	0.05 (-0.20; 0.29)	0.14 (-0.11; 0.39)	0.1192	0.6791	0.2600	0.2687
VOLT Speed	0.06 (-0.22; 0.35)	0.08 (-0.21; 0.36)	-0.44 (-0.72; -0.16)	0.6389	0.5656	0.0036*	0.01 (-0.40; 0.43)	-0.51 (-0.90; -0.11)	0.52 (0.12; 0.92)	0.9407	0.0154	0.0133	0.0185
F2B Speed	0.08 (-0.25; 0.40)	-0.32 (-0.64; 0.00)	0.14 (-0.18; 0.45)	0.6166	0.0512	0.3719	-0.40 (-0.86; 0.07)	0.06 (-0.40; 0.51)	-0.45 (-0.90; -0.01)	0.0883	0.7927	0.0472	0.1005
AM Speed	0.08 (-0.24; 0.40)	0.08 (-0.25; 0.40)	-0.11 (-0.42; 0.21)	0.6255	0.6300	0.4893	0.00 (-0.47; 0.47)	-0.18 (-0.63; 0.27)	0.18 (-0.28; 0.64)	0.9993	0.4045	0.4150	0.6228
LOT Speed	0.16 (-0.27; 0.60)	-0.12 (-0.55; 0.31)	-0.30 (-0.74; 0.13)	0.4414	0.5623	0.1602	-0.28 (-0.90; 0.33)	-0.47 (-1.09; 0.15)	0.18 (-0.43; 0.80)	0.3446	0.1313	0.5435	0.3048
ERT Speed	-0.59 (-0.91; -0.27)	-0.48 (-0.79; -0.16)	-0.57 (-0.89; -0.25)	0.0004**	0.0037*	0.0006*	0.11 (-0.33; 0.56)	0.02 (-0.44; 0.47)	0.10 (-0.35; 0.55)	0.6123	0.9401	0.6686	0.8598
MRT Speed	-0.20 (-0.65; 0.25)	-0.36 (-0.79; 0.07)	-0.55 (-0.98; -0.12)	0.3692	0.0989	0.0143	-0.16 (-0.80; 0.48)	-0.36 (-0.99; 0.28)	0.20 (-0.40; 0.79)	0.6082	0.2575	0.5023	0.5108
DSST Speed	0.04 (-0.10; 0.18)	-0.01 (-0.15; 0.14)	0.12 (-0.03; 0.26)	0.5605	0.9397	0.1062	-0.05 (-0.25; 0.16)	0.08 (-0.13; 0.28)	-0.12 (-0.32; 0.08)	0.6429	0.4454	0.2268	0.4652
BART Speed	-0.13 (-0.42; 0.16)	-0.48 (-0.78; -0.17)	-0.20 (-0.49; 0.10)	0.3638	0.0041*	0.1740	-0.35 (-0.78; 0.08)	-0.07 (-0.48; 0.34)	-0.28 (-0.72; 0.16)	0.1080	0.7285	0.1951	0.2403
PVT Speed	0.14 (-0.24; 0.52)	-0.02 (-0.39; 0.35)	0.22 (-0.16; 0.60)	0.4396	0.9182	0.2380	-0.16 (-0.70; 0.37)	0.08 (-0.47; 0.63)	-0.24 (-0.77; 0.29)	0.5337	0.7719	0.3581	0.6337
MP Accuracy	0.21 (-0.35; 0.77)	0.13 (-0.42; 0.67)	0.37 (-0.21; 0.94)	0.4310	0.6268	0.1972	-0.09 (-0.86; 0.69)	0.15 (-0.68; 0.98)	-0.24 (-1.04; 0.57)	0.8185	0.7089	0.5446	0.8271
VOLT Accuracy	-0.03 (-0.50; 0.43)	-0.02 (-0.47; 0.43)	0.22 (-0.23; 0.67)	0.8840	0.9379	0.3115	0.02 (-0.65; 0.68)	0.26 (-0.41; 0.92)	-0.24 (-0.87; 0.39)	0.9605	0.4283	0.4341	0.6502
F2B Accuracy	-0.21 (-0.83; 0.42)	-0.05 (-0.68; 0.57)	-0.39 (-1.01; 0.23)	0.4932	0.8583	0.2007	0.15 (-0.74; 1.04)	-0.18 (-1.07; 0.70)	0.34 (-0.54; 1.22)	0.7210	0.6652	0.4309	0.7260
AM Accuracy	-0.17 (-0.40; 0.06)	-0.01 (-0.24; 0.22)	0.19 (-0.04; 0.42)	0.1453	0.9255	0.1027	0.16 (-0.17; 0.48)	0.36 (0.03; 0.69)	-0.20 (-0.53; 0.13)	0.3353	0.0310	0.2233	0.0950
LOT Accuracy	-0.06 (-0.57; 0.45)	0.20 (-0.30; 0.71)	0.25 (-0.26; 0.75)	0.8047	0.4070	0.3183	0.27 (-0.46; 0.99)	0.31 (-0.41; 1.03)	-0.04 (-0.76; 0.67)	0.4501	0.3805	0.9036	0.6317
ERT Accuracy	0.04 (-0.29; 0.37)	-0.08 (-0.41; 0.26)	-0.16 (-0.50; 0.18)	0.8119	0.6371	0.3344	-0.11 (-0.58; 0.36)	-0.20 (-0.68; 0.28)	0.08 (-0.40; 0.56)	0.6161	0.4001	0.7183	0.6918
MRT Accuracy	-0.21 (-0.65; 0.23)	0.27 (-0.17; 0.71)	0.16 (-0.28; 0.60)	0.3330	0.2089	0.4503	0.48 (-0.15; 1.11)	0.37 (-0.25; 0.99)	0.11 (-0.51; 0.73)	0.1250	0.2286	0.7073	0.2684
DSST Accuracy	0.22 (-0.06; 0.50)	-0.04 (-0.33; 0.26)	0.24 (-0.02; 0.51)	0.1230	0.8034	0.0732	-0.25 (-0.69; 0.18)	0.02 (-0.35; 0.40)	-0.28 (-0.68; 0.13)	0.2416	0.8957	0.1712	0.3494
BART Risk Taking	0.23 (-0.31; 0.78)	0.31 (-0.28; 0.90)	0.19 (-0.40; 0.78)	0.3815	0.2837	0.4985	0.07 (-0.73; 0.88)	-0.04 (-0.85; 0.77)	0.12 (-0.77; 1.00)	0.8473	0.9175	0.7880	0.9621
PVT Accuracy	0.09 (-0.34; 0.51)	-0.17 (-0.58; 0.25)	0.06 (-0.35; 0.48)	0.6749	0.4072	0.7561	-0.25 (-0.85; 0.35)	-0.02 (-0.62; 0.57)	-0.23 (-0.81; 0.35)	0.3864	0.9355	0.4190	0.6178
Speed	-0.06 (-0.21; 0.09)	-0.13 (-0.28; 0.02)	-0.18 (-0.33; -0.03)	0.4239	0.0786	0.0197	-0.07 (-0.29; 0.14)	-0.12 (-0.34; 0.09)	0.05 (-0.16; 0.26)	0.4772	0.2416	0.6344	0.4909
Accuracy	-0.03 (-0.20; 0.13)	0.08 (-0.09; 0.25)	0.07 (-0.10; 0.24)	0.6667	0.3160	0.3995	0.12 (-0.12; 0.36)	0.10 (-0.13; 0.34)	0.01 (-0.23; 0.26)	0.3139	0.3693	0.9056	0.5315
Efficiency	-0.05 (-0.16; 0.06)	-0.03 (-0.14; 0.08)	-0.05 (-0.16; 0.06)	0.3319	0.6291	0.3356	0.03 (-0.13; 0.18)	0.00 (-0.15; 0.15)	0.03 (-0.13; 0.18)	0.7301	0.9899	0.7355	0.9240
Sleep Duration [h]	-0.07 (-0.41; 0.27)	-0.15 (-0.49; 0.19)	-0.57 (-0.91; -0.24)	0.6769	0.3869	0.0009**	0.03 (-0.50; 0.57)	-0.47 (-1.00; 0.05)	0.51 (-0.02; 1.03)	0.8950	0.0748	0.0583	0.1026
Poor Sleep Quality	0.0 (-1.1; 1.1)	0.2 (-1.0; 1.3)	-1.0 (-2.1; 0.1)	1.0000	0.7744	0.0863	0.2 (-2.0; 2.4)	-0.7 (-2.9; 1.4)	1.0 (-1.3; 3.2)	0.8352	0.4784	0.3907	0.6532
Low Workload	-1.2 (-2.2; -0.2)	-1.7 (-2.7; -0.7)	-0.8 (-1.8; 0.3)	0.0194*	0.0010**	0.1457	-0.7 (-2.4; 0.9)	0.2 (-1.4; 1.8)	-0.9 (-2.5; 0.6)	0.3501	0.8097	0.2223	0.4311
Sleepy	1.7 (0.7; 2.6)	1.3 (0.3; 2.2)	-0.3 (-1.3; 0.7)	0.0009**	0.0123*	0.5571	-0.4 (-2.2; 1.4)	-1.8 (-3.6; -0.1)	1.4 (-0.3; 3.2)	0.6298	0.0421	0.1015	0.0964
Unhappy	0.1 (-0.8; 1.1)	1.0 (0.1; 1.9)	0.5 (-0.4; 1.5)	0.7949	0.0383	0.2605	0.7 (-1.0; 2.3)	0.4 (-1.3; 2.1)	0.3 (-1.4; 1.9)	0.4156	0.6292	0.7344	0.7091
Healthy	-3.4 (-4.5; -2.3)	-1.9 (-3.0; -0.8)	-2.0 (-3.1; -0.8)	<.0001****	0.0009**	0.0007**	2.1 (-0.2; 4.4)	1.0 (-1.4; 3.4)	1.0 (-1.3; 3.4)	0.0761	0.3771	0.3700	0.1985
Physically Exhausted	2.8 (1.8; 3.9)	1.7 (0.7; 2.8)	1.5 (0.4; 2.5)	<.0001****	0.0016**	0.0070*	-1.0 (-3.3; 1.2)	-1.1 (-3.3; 1.2)	0.0 (-2.1; 2.1)	0.3382	0.3220	0.9638	0.5374
Mentally Fatigued	1.4 (0.4; 2.4)	1.9 (0.9; 2.9)	0.5 (-0.5; 1.5)	0.0072*	0.0003**	0.3259	0.4 (-1.9; 2.7)	-0.8 (-3.1; 1.5)	1.2 (-1.1; 3.5)	0.7226	0.4688	0.2839	0.5414
Stressed	2.3 (1.2; 3.3)	2.7 (1.6; 3.7)	0.8 (-0.2; 1.9)	<.0001***	<.0001****	0.1140	0.1 (-2.2; 2.4)	-0.5 (-3.0; 2.0)	0.6 (-1.9; 3.1)	0.9280	0.6694	0.6063	0.8619
Fresh	-1.5 (-2.5; -0.5)	-1.8 (-2.8; -0.7)	-0.6 (-1.6; 0.4)	0.0040*	0.0008**	0.2599	-0.1 (-1.6; 1.4)	0.8 (-0.7; 2.3)	-0.9 (-2.4; 0.6)	0.8750	0.2595	0.2064	0.3787
Not Depressed	-0.7 (-1.6; 0.3)	-1.9 (-2.8; -0.9)	-1.5 (-2.4; -0.6)	0.1594	0.0001***	0.0017**	-0.9 (-2.7; 0.9)	-0.8 (-2.6; 1.0)	-0.1 (-1.9; 1.7)	0.3023	0.3777	0.8745	0.5325
Not Bored	-0.3 (-1.2; 0.5)	-0.5 (-1.4; 0.3)	-0.3 (-1.2; 0.5)	0.4519	0.2219	0.4519	-0.1 (-1.3; 1.2)	-0.1 (-1.4; 1.2)	0.0 (-1.2; 1.3)	0.9094	0.8622	0.9506	0.9841
Lonely	0.3 (-0.6; 1.3)	1.3 (0.3; 2.3)	1.7 (0.7; 2.6)	0.5029	0.0098*	0.0009**	0.7 (-1.3; 2.7)	1.4 (-0.6; 3.4)	-0.7 (-2.6; 1.3)	0.4597	0.1577	0.4835	0.3584
Not Monotonous	-1.3 (-2.3; -0.3)	-0.5 (-1.4; 0.5)	-0.4 (-1.4; 0.6)	0.0106*	0.3619	0.4072	0.8 (-0.6; 2.2)	0.9 (-0.6; 2.3)	-0.1 (-1.5; 1.3)	0.2550	0.2180	0.9302	0.3867

All models were adjusted for sex and age. Models with cognitive outcomes were additionally adjusted for baseline performance. Estimates for cognitive tests reflect z-scores. As z-transformation was based on baseline performance, an estimate of 0 (zero) reflects baseline performance. Estimates for self-report data reflect points on an 11-point scale (variables are listed by anchors for high values). As sleep and subjective outcomes were not z-transformed, estimates for these variables were not adjusted for baseline values but are based on a direct contrast between the head-down tilt and baseline period instead. Est.: Estimate; CI: Confidence Interval; CTRL: Control; cAG: continuous Artificial Gravity; iAG: intermittent Artificial Gravity; *adjusted p<0.05; **adjusted p<0.01; ***adjusted p<0.001; ****adjusted p<0.0001; MP: Motor Praxis; VOLT: Visual Object Learning Test; F2B: Fractal 2-Back; AM: Abstract Matching; LOT: Line Orientation Test; ERT: Emotion Recognition Test; MRT: Matrix Reasoning Test; DSST: Digit Symbol Substitution Test; BART: Balloon Analog Risk Test; PVT: Psychomotor Vigilance Test

Table S4: Mixed effect model results for time in head-down tilt bed rest analyses

	Pooled		Control		Continuous AG		Intermittent AG		DiHDT*Intervention
**Variable**	**Change per DiHDT**	**p-value**	**Change per DiHDT**	**p-value**	**Change per DiHDT**	**p-value**	**Change per DiHDT**	**p-value**	**p-value**
MP Speed	0.002 (0.002)	0.1853	0.005 (0.002)	0.0061	0.001 (0.002)	0.5782	0.000 (0.004)	0.9276	0.4307
VOLT Speed	-0.003 (0.002)	0.2041	-0.001 (0.003)	0.8527	-0.001 (0.004)	0.7575	-0.006 (0.004)	0.1569	0.4993
F2B Speed	0.004 (0.003)	0.1365	0.011 (0.006)	0.1072	0.001 (0.004)	0.7558	0.000 (0.003)	0.9737	0.1847
AM Speed	0.000 (0.003)	0.8968	0.001 (0.006)	0.8933	0.003 (0.004)	0.4947	-0.005 (0.003)	0.0917	0.4573
LOT Speed	0.000 (0.003)	0.9470	0.003 (0.005)	0.6025	0.004 (0.003)	0.2132	-0.007 (0.006)	0.2730	0.2217
ERT Speed	-0.009 (0.002)	0.0001**	-0.007 (0.003)	0.0138	-0.007 (0.003)	0.0205	-0.014 (0.005)	0.0185	0.2580
MRT Speed	-0.003 (0.003)	0.2756	-0.007 (0.003)	0.0135	0.004 (0.006)	0.5882	-0.005 (0.004)	0.2171	0.2234
DSST Speed	0.003 (0.002)	0.0710	0.002 (0.001)	0.1238	0.004 (0.002)	0.0788	0.004 (0.005)	0.4457	0.8726
BART Speed	0.001 (0.003)	0.6513	0.005 (0.003)	0.1013	0.003 (0.006)	0.6536	-0.004 (0.003)	0.2420	0.2958
PVT Speed	0.004 (0.003)	0.1162	0.007 (0.005)	0.2035	0.006 (0.003)	0.0459	-0.001 (0.006)	0.9192	0.4382
MP Accuracy	-0.006 (0.003)	0.0331	-0.004 (0.005)	0.4174	-0.004 (0.006)	0.5650	-0.011 (0.005)	0.0477	0.5379
VOLT Accuracy	0.007 (0.003)	0.0318	0.008 (0.007)	0.2272	0.006 (0.006)	0.3033	0.007 (0.005)	0.2197	0.9584
F2B Accuracy	-0.004 (0.003)	0.1744	-0.002 (0.004)	0.6382	-0.001 (0.007)	0.8722	-0.009 (0.005)	0.0718	0.4908
AM Accuracy	0.001 (0.002)	0.5794	0.000 (0.005)	0.9825	-0.002 (0.003)	0.5665	0.006 (0.005)	0.2567	0.4108
LOT Accuracy	0.001 (0.003)	0.7726	0.007 (0.006)	0.3025	-0.004 (0.004)	0.2915	0.001 (0.006)	0.9334	0.3329
ERT Accuracy	0.001 (0.003)	0.7430	-0.001 (0.006)	0.8041	-0.002 (0.005)	0.7302	0.007 (0.007)	0.3536	0.5131
MRT Accuracy	0.001 (0.004)	0.7373	0.007 (0.005)	0.1743	0.003 (0.008)	0.7396	-0.006 (0.006)	0.3370	0.3527
DSST Accuracy	0.002 (0.003)	0.5319	0.000 (0.004)	0.9791	0.006 (0.006)	0.3721	-0.001 (0.004)	0.8944	0.6093
BART Risk Taking	0.002 (0.003)	0.4592	-0.001 (0.006)	0.9089	0.003 (0.004)	0.3632	0.005 (0.007)	0.5433	0.7911
PVT Accuracy	0.004 (0.005)	0.4553	0.013 (0.012)	0.2882	0.004 (0.004)	0.2798	-0.005 (0.011)	0.6465	0.3994
Speed	0.000 (0.001)	0.9538	0.002 (0.001)	0.1188	0.002 (0.002)	0.3315	-0.004 (0.002)	0.0482	0.0227
Accuracy	0.001 (0.001)	0.5039	0.003 (0.002)	0.2075	0.001 (0.002)	0.5963	-0.001 (0.003)	0.6554	0.3719
Efficiency	0.000 (0.001)	0.6661	0.002 (0.001)	0.1017	0.001 (0.001)	0.1385	-0.003 (0.002)	0.2110	0.0488
Sleep Duration [h]	0.001 (0.002)	0.6458	0.002 (0.007)	0.7911	0.003 (0.003)	0.3077	-0.002 (0.003)	0.5654	0.6995
Poor Sleep Quality	0.011 (0.008)	0.1819	0.022 (0.015)	0.1812	-0.003 (0.012)	0.7790	0.014 (0.014)	0.3608	0.4159
Low Workload	0.01 (0.007)	0.1546	-0.001 (0.012)	0.9458	0.011 (0.015)	0.4897	0.021 (0.012)	0.0892	0.4656
Sleepy	-0.001 (0.007)	0.8429	0.002 (0.014)	0.8732	-0.007 (0.011)	0.5712	0.000 (0.012)	0.9718	0.8598
Unhappy	0.009 (0.008)	0.2635	0.003 (0.018)	0.8659	0.004 (0.01)	0.6780	0.019 (0.013)	0.2026	0.6528
Healthy	-0.005 (0.007)	0.4379	-0.015 (0.014)	0.3028	-0.003 (0.012)	0.8022	0.001 (0.01)	0.8872	0.6186
Physically Exhausted	0.005 (0.007)	0.4556	0.007 (0.015)	0.6302	-0.002 (0.014)	0.8685	0.011 (0.01)	0.2735	0.7437
Mentally Fatigued	0.004 (0.007)	0.5461	-0.007 (0.01)	0.4912	0.002 (0.015)	0.8879	0.018 (0.009)	0.1000	0.3480
Stressed	0.004 (0.006)	0.4758	0.012 (0.012)	0.3203	-0.006 (0.009)	0.5255	0.006 (0.008)	0.4567	0.4329
Fresh	0.000 (0.007)	0.9553	0.004 (0.016)	0.7893	0.001 (0.014)	0.9681	-0.006 (0.01)	0.5258	0.8526
Not Depressed	-0.012 (0.008)	0.1346	0.006 (0.011)	0.5718	-0.016 (0.012)	0.2320	-0.026 (0.016)	0.1417	0.2233
Not Bored	-0.008 (0.007)	0.2667	-0.011 (0.01)	0.3083	-0.006 (0.019)	0.7463	-0.007 (0.015)	0.6274	0.9726
Lonely	0.006 (0.008)	0.4242	0.007 (0.016)	0.6490	-0.004 (0.009)	0.6983	0.016 (0.016)	0.3583	0.6235
Not Monotonous	-0.022 (0.009)	0.0256	-0.024 (0.017)	0.1931	-0.009 (0.011)	0.4484	-0.033 (0.02)	0.1447	0.5818
ERT Happy	-0.01 (0.004)	0.0163*	-0.015 (0.007)	0.0812	-0.013 (0.007)	0.1687	-0.003 (0.007)	0.7111	0.4259
ERT Sad	0.004 (0.003)	0.0915	0.011 (0.005)	0.0830	0.002 (0.003)	0.6280	0.001 (0.006)	0.8910	0.2516
ERT Angry	0.012 (0.003)	0.0006**	0.01 (0.005)	0.0363	0.004 (0.004)	0.3927	0.022 (0.006)	0.0010*	0.0306
ERT Fear	0.002 (0.004)	0.5808	-0.005 (0.006)	0.3971	0.003 (0.005)	0.6309	0.012 (0.008)	0.1715	0.1940
ERT Neutral	-0.008 (0.003)	0.0074*	-0.006 (0.003)	0.0999	0.000 (0.004)	0.9356	-0.016 (0.005)	0.0010*	0.0243

All models were adjusted for sex, age and baseline values. Estimates for cognitive tests reflect z-scores. Estimates for self-report data reflect points on an 11-point scale (variables are listed by anchors for high values). DiHDT: Day in head-down tilt; Change per DiHDT reflects estimate (standard error). DiHDT*Intervention reflects a test for the interaction between DiHDT (continuous) and the three intervention groups (Control, cAG, iAG). "Pooled" reflects an analysis with data pooled across the three experimental groups. ERT Happy/Sad/Angry/Fear/Neutral expresses the tendency to rate an item in the respective category based on comparisons to responses of a normative group of subjects. Adjustments for ERT tendency p-values were based on N=5 comparisons. *adjusted $p<0.05$; **adjusted $p<0.01$; AG: Artificial Gravity; MP: Motor Praxis; VOLT: Visual Object Learning Test; F2B: Fractal 2-Back; AM: Abstract Matching; LOT: Line Orientation Test; ERT: Emotion Recognition Test; MRT: Matrix Reasoning Test; DSST: Digit Symbol Substitution Test; BART: Balloon Analog Risk Test; PVT: Psychomotor Vigilance Test
